# Whole-genome sequence of the oriental lung fluke *Paragonimus westermani*

**DOI:** 10.1093/gigascience/giy146

**Published:** 2018-12-06

**Authors:** Harald Oey, Martha Zakrzewski, Kanwar Narain, K Rekha Devi, Takeshi Agatsuma, Sujeevi Nawaratna, Geoffrey N Gobert, Malcolm K Jones, Mark A Ragan, Donald P McManus, Lutz Krause

**Affiliations:** 1 The University of Queensland Diamantina Institute, Faculty of Medicine, The University of Queensland, 37 Kent St, Translational Research Institute (TRI), Wooloongabba, QLD 4102; 2 Molecular Parasitology Laboratory, Immunology Department, QIMR Berghofer Medical Research Institute, 300 Herston Road, QLD 4006, Australia; 3 School of Veterinary Science, University of Queensland, Gatton, QLD 4343, Australia; 4 School of Biological Sciences, Queen's University Belfast, 19 Chlorine Gardens, Belfast BT9 5DL, United Kingdom; 5 Institute for Molecular Bioscience, The University of Queensland, 306 Carmody Road, St Lucia, QLD 4072, Australia; 6 ICMR-Regional Medical Research Centre, Dibrugarh - 786010, Assam, India; 7 Department of Environmental Medicine, Kochi University, Kohasu, Oko, Nankoku City 783–8505, Japan; 8 School of Medicine, Griffith University, Gold Coast Campus, QLD 4222, Australia

**Keywords:** *Paragonimus westermani*, whole-genome sequence, genome assembly, paragonimiasis, food-borne disease, oriental lung fluke, parasitic infection, comparative genomics, neglected tropical disease, flatworm

## Abstract

**Background:**

Foodborne infections caused by lung flukes of the genus *Paragonimus* are a significant and widespread public health problem in tropical areas. Approximately 50 *Paragonimus* species have been reported to infect animals and humans, but *Paragonimus westermani* is responsible for the bulk of human disease. Despite their medical and economic importance, no genome sequence for any *Paragonimus* species is available.

**Results:**

We sequenced and assembled the genome of *P. westermani*, which is among the largest of the known pathogen genomes with an estimated size of 1.1 Gb. A 922.8 Mb genome assembly was generated from Illumina and Pacific Biosciences (PacBio) sequence data, covering 84% of the estimated genome size. The genome has a high proportion (45%) of repeat-derived DNA, particularly of the long interspersed element and long terminal repeat subtypes, and the expansion of these elements may explain some of the large size. We predicted 12,852 protein coding genes, showing a high level of conservation with related trematode species. The majority of proteins (80%) had homologs in the human liver fluke *Opisthorchis viverrini*, with an average sequence identity of 64.1%. Assembly of the *P. westermani* mitochondrial genome from long PacBio reads resulted in a single high-quality circularized 20.6 kb contig. The contig harbored a 6.9 kb region of non-coding repetitive DNA comprised of three distinct repeat units. Our results suggest that the region is highly polymorphic in *P. westermani*, possibly even within single worm isolates.

**Conclusions:**

The generated assembly represents the first *Paragonimus* genome sequence and will facilitate future molecular studies of this important, but neglected, parasite group.

## Background

*Paragonimus* lung flukes represent a significant and widespread clinical problem, with an estimated 23 million people infected worldwide [1]. Approximately 50 species are described, with at least 7 being human pathogens [2]. The majority of human *Paragonimus* infections can be attributed to the *Paragonimus westermani* species complex, mainly in Southeast Asia and Japan [1]. *Paragonimus westermani* show considerable geographic genetic variability, and human infections occur predominantly in East Asia and the Philippines. In India the incidence rates of paragonimiasis caused by *P. westermani* is currently unknown [2–4]; however, many cases of paragonimiasis are attributed to the related worm *Paragonimus heterotremus* [2]. Paragonimiasis is a zoonotic disease; pigs, dogs, and other animals can also harbor *P. westermani* [2].

*Paragonimus* spp. have a complex life cycle. Unembryonated eggs are expelled by coughing or passed with stool and develop in water. Miracidia hatch from the eggs and penetrate a freshwater snail, its first intermediate host. During several asexual developmental phases inside the snail, a miracidium develops into a sporocyst and then two redial generations occur, the second of which gives rise to microcercous cercariae that escape into fresh water. These crawling cercariae invade a species of crustacean, the second intermediate host, to encyst in muscles and other sites and develop into metacercariae. Humans and other definitive hosts become infected through consumption of raw or inadequately cooked freshwater crabs or crayfish [5]. Ingested metacercariae excyst, penetrate through the gut, and become encapsulated in the lungs where they mature into hermaphroditic adult worms (7.5 mm to 12 mm in length) in 6–10 weeks [5]. Paragonimiasis can lead to a chronic inflammatory disease of the lung and can trigger asthma- or tuberculosis-like symptoms [6–8]. In more severe cases, *Paragonimus* can infect the brain or central nervous system of the definitive host, leading to headache, visual loss, and even death [1].

Paragonimiasis is commonly diagnosed by microscopic detection of parasite eggs in stool or sputum. The lack of sensitive and reliable diagnostic tests in conjunction with unspecific disease symptoms often leads to delayed treatment with the drug of choice, praziquantel [8]. Despite their high medical, veterinary, and economic importance, only limited information on the molecular biology of *Paragonimus* is currently available. Recent transcriptome sequencing studies have provided some information on the gene content of *Paragonimus* [9]; however, to date, no *Paragonimus* genome sequence has been available. Here, we present a 922.8 Mb assembly of the *P. westermani* genome that provides new insights into the genomic composition of the *Paragonimus* genus and represents an invaluable resource for future studies of the neglected tropical disease paragonimiasis.

## Data Description

### Sequencing

**Figure 1:**
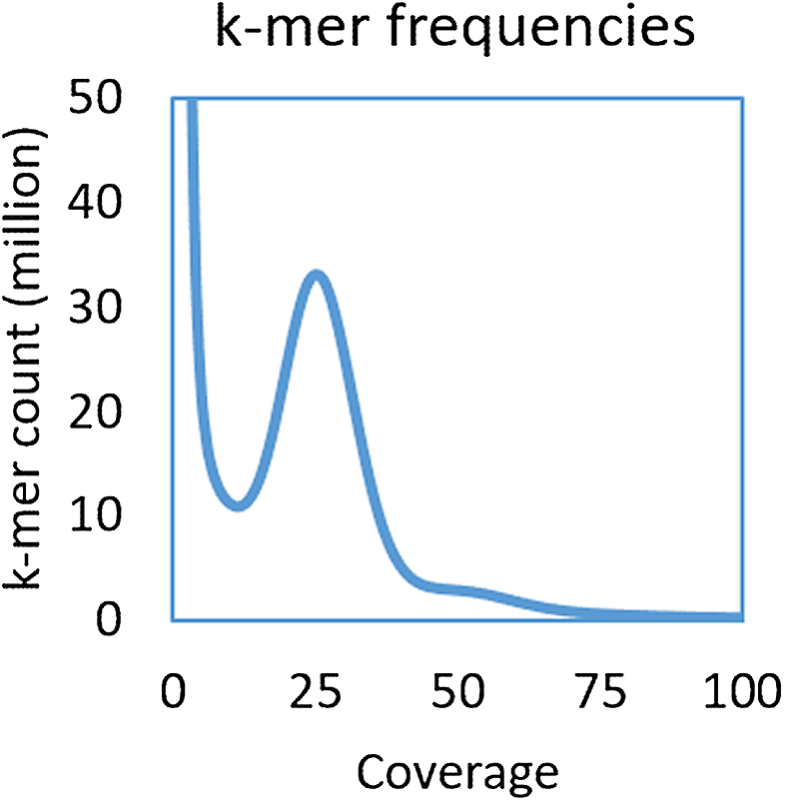
*k*-mer frequencies for the 450 bp library. Distribution of 17-mers in the 450 bp short-insert library demonstrated low sequence heterozygosity. We observed a single peak at 26×, and the *P. westermani* genome size was estimated to be 1.1 Gb.

Diploid *P. westermani* metacercariae (National Center for Biotechnology Information [NCBI]: txid34504) were collected from the freshwater crab *Maydelliathelphusa lugubris* in 2009 in the Changlang District of Arunachal Pradesh, northeast India, and fed to Wistar rats as experimental hosts. Genomic DNA was isolated from a pool of 50 worms (30–40 days of age), yielding 18 µg of DNA. DNA was quantified by Pico green, Qubit, and NanoDrop; degradation was tested by microplate reader and agarose gel electrophorese (concentration of agarose gel, 1%; electrophoresis time, 40 minutes; voltage, 150 V). The *P. westermani* genome was then sequenced from 2 µg of the isolated DNA using a whole-genome shotgun approach. Paired-end short-insert (200 bp and 450 bp) and mate-pair (5 kb and 10 kb) genomic DNA libraries were sequenced on the Illumina HiSeq 2000 platform, yielding 58 Gb of sequence data (Table 1). For genome scaffolding and quality evaluation of the assembled sequence, additional long-read data were generated from the same genomic DNA sample using the Pacific Biosciences (PacBio) RSII platform, yielding 1.7 Gb of information (Table 1). The genome size was estimated from the *k*-mer coverage of the 450 bp insert library. *k*-mer frequencies were calculated by the program Jellyfish [10], version 2.2.6, using a *k*-mer size of 17 bp. The 17-mer distribution in the 450 bp library had a single peak at 26× (Fig. 1), demonstrating low sequence heterozygosity. The genome size (G) was deduced from the *k*-mer distribution via the formula G = N * (L–K + 1)/K_depth [11], where N is the total number of reads, L is the read length, K is the *k*-mer size and K_depth is the peak frequency. The *P. westermani* genome size was estimated to be 1.1 Gb.

**Table 1:** *Paragonimus westermani* sequencing libraries

Library	Platform	Library type	Insert size (bp)	Read length (bp)	Read count (raw)
200 bp	HiSeq	Paired-end	200	2 × 120	140,542,299
450 bp	HiSeq	Paired-end	450	2 × 100	171,954,230
5kb	HiSeq	Mate-pair	5000	2 × 49	232,630,904
10kb	HiSeq	Mate-pair	10 000	2 × 49	266,480,540
PacBio	PacBio	Long read	–	–	1,731,327

### Genome assembly

PacBio sequence data were error corrected by proovread version 2.13.13 [12], using Illumina short reads from the 200 bp and 450 bp libraries as input, and assembled into contigs by Mira v4.0.2 (MIRA, RRID:SCR_010731) [13]. Short-read Illumina sequence data were trimmed using Trimmomatic v0.36 (Trimmomatic, RRID:SCR_011848) and subsequently error corrected by KmerFreq_HA (part of SoapDenovo2 [14]) with a *k*-mer size of 23. The 10 kb mate-pair library showed a high proportion of polymerase chain reaction (PCR) duplicates and was subjected to PCR de-duplication prior to genome assembly. For assembly of short-read data, several assembly programs were evaluated. ABYSS performed best for this particular genome with its large size, high percentage of repetitive regions, and some low-level sequence heterogeneity resulting from pooling genomic DNA from the 50 individual worms. ABYSS is also one of the few assemblers that allows inclusion of long-read data to guide scaffolding. Illumina paired-end sequence data were assembled using the ABYSS assembly pipeline (ABySS, RRID:SCR_010709) [15], version 2.0.2, with options n = 5 s = 200 N = 36 S = 500 k = 33 and including the PacBio contigs via the re-scaffolding feature.

The resulting assembly was de-gapped using the SoapDenovo2 GapCloser program (GapCloser, RRID:SCR_015026) [14], which is well suited for closing gaps larger than 1kb; it performed particularly well on this genome. Mate-pair libraries were then used to scaffold the assembly with SSPACE v3.0 [16] (with options −x 0 −a 0.60 −n 30 −z 200 −g 0) and gaps were again filled with GapCloser. Un-closed gaps are represented by Ns spanning the estimated sizes of the gaps. To detect and resolve scaffolding errors, the resulting assembly was processed by the program REAPR [17] using the 5 kb mate-pair library as input, breaking the assembly at sites with poor evidence for contiguity. Contamination due to the experimental rat host and the bacterium *Delftia* sp. was detected based on a comparison of predicted proteins with the NCBI protein database using the Basic Local Alignment Search Tool (BLAST) and, additionally, via the NCBI Genome Submission Portal quality control pipeline. A targeted comparison of all scaffolds with the genomes of the rat and *Delftia* using the BLAST-like alignment tool identified 531 short scaffolds with high similarity (>90%) to these genomes. These sequences were manually scrutinized, with 529 of the affected scaffolds found to be completely derived from rat or *Delftia*, and these were removed from the assembly. The remaining two contaminated sequences represented rat ribosomal DNA that had been erroneously incorporated into *Paragonimus* scaffolds and were also removed from the final assembly by cutting and trimming the affected scaffolds.

The final assembly resulted in a 922.8 Mb genome sequence (30,466 scaffolds with N50 of 135 kb) (Table 2), covering 84.0% of the estimated genome size. The discrepancies in genome size can potentially be the result of problematic DNA regions that are difficult to sequence or assemble (e.g., regions with strong secondary structures, highly repetitive regions, or long homopolymeric runs) or the result of low-level sequence heterogeneity, which can lead to an overestimation of genome size by *k*-mer approaches. The *P. westermani* genome sequence is among the largest known pathogen genomes and one of the largest parasite genomes sequenced to date. The assembled genome sequence is considerably larger than the published genomes of the related trematodes *Clonorchis sinensis* (assembly size of 546.9 Mb) [18], *Opisthorchis viverrini* (606.0 Mb) [19], and *Schistosoma* spp. (364.5–397.7 Mb) [20–22] and comparable to the 1.3 Gb genome of *Fasciola hepatica* [23].

**Table 2:** Assembly statistics for *P. westermani* and comparable trematode genomes of similar size

	*P. westermani*	*F. hepatica*	*O. viverrini*	*C. sinensis*
Assembly size (Mb)^a^	922.8	1,275.0	606.0	546.9
Ungapped size (Mb)^b^	877.7	1,183.5	558.0	547.1
Contig N50 (kb)	7.0 (>100 bp)	9.7	NA	14.7
Scaffold N50 (kb)	135 (>1kb)	204	1,324	30.2
Scaffold L50	1943	1,799	135	408
Scaffold count	30,466 (>1 kb)	45,354 (>1 kb)	4,919 (>1kb)	31,822
GC content (%)	43.3	44.1	43.8	44.1
Repeat content (%)	45.2	57.1	28.9	32.6
Protein coding genes	12,852	15,740^c^	16,356	13,634
Longest scaffold (kb)	809	1,565	9,657	2050
BUSCO—Complete	65.3%	65.8%	71.4%	70.8%
BUSCO—Duplicated	1.4%	0.8%	1.1%	1.5%
BUSCO—Missing	25.8%	25.4%	23.0%	23.1%

a Combined length of all scaffolds in Mb.

b Combined length of all scaffolds without gaps (Ns) in Mb.

c Non-overlapping RNA-sequencing-supported gene models [23]. BUSCO: Benchmarking Universal Single-Copy Orthologs.

The GC content of the genome was 43.3%, comparable to genomes of other related trematodes (Table 2). Genome assembly completeness was evaluated by Benchmarking Universal Single-Copy Orthologs (BUSCO) (BUSCO, RRID:SCR_015008) [24] using the metazoan lineage data, resulting in scores similar to those obtained for the genomes of comparable trematode species (Table 2). The proportion of duplicated genes reported by BUSCO was also similar to that of comparable trematodes, suggesting that the relatively large size of the *P. westermani* genome is not the result of genome duplication events.

### Mitochondrial genome

**Figure 2:**
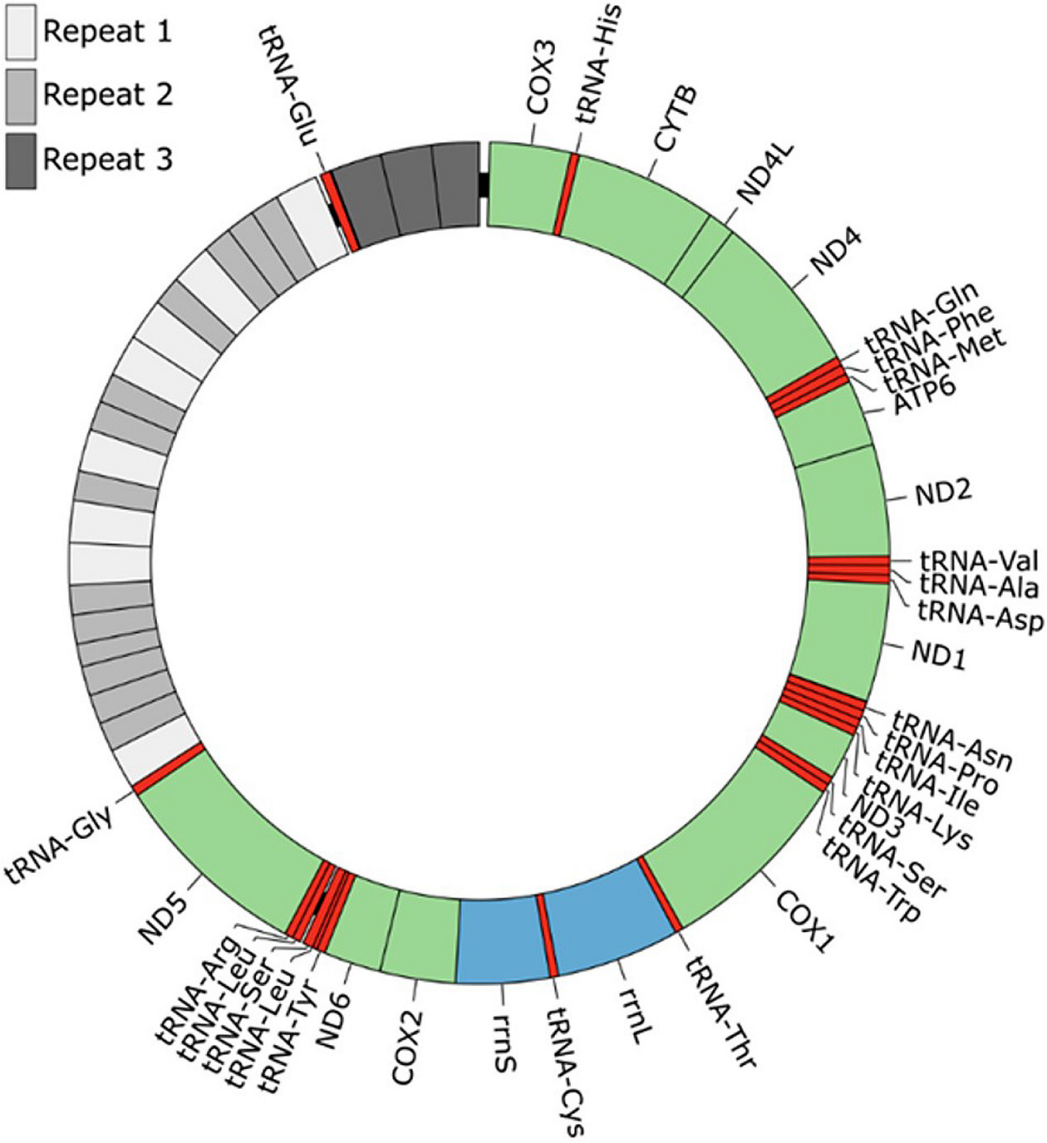
The complete *P. westermani* mitochondrial genome. A graphical representation of the *P. westermani* circular mitochondrial genome is shown, including an ∼6.9 kb repetitive region. Three distinct repeat units were identified in this region, as well as an intervening tRNA gene (tRNA-Glu). All genes are transcribed in the clock-wise direction.

The mitochondrial genome of *P. westermani* is present at a much higher copy number than the nuclear genome, and we were able to assemble the full mitochondrial genome at high coverage from error-corrected long PacBio reads using the Mira assembler [13], version 4.0.2. This resulted in a single mitochondrial contig of 20.3 kb (Fig. 2). The accuracy of the contig was confirmed by mapping short insert paired-end sequences directly onto the contig, revealing single nucleotide discrepancies at only four positions. The mitochondrial genome was found to closely match previously published *Paragonimus* mitochondrial genomes, with the best match from a BLAST search against the Nucleotide collection at the NCBI [25] being accession NC_027673.1, a *P. westermani* complex sp. type 1 mitochondrial genome isolated in India (97% sequence identity across 13.4 kb of NC_027673.1). This sequence was used as reference for mitochondrial gene identification and annotation, supplemented by mitochondrial gene predictions by Mitos [26] and tRNA prediction by Aragorn (Aragorn, RRID:SCR_015974) [27]. The mitochondrial genomes of flatworms are known to harbor a region of non-coding repetitive DNA, generally comprised of a long non-coding region (LNR) and a short non-coding region (SNR) with a single tRNA gene separating them [28]. Reconstructing this region from short-read data proved challenging, but our long-read PacBio data allowed complete assembly of the repetitive region and circularization of the genome. Interestingly, our assembled mitochondrial genome sequence had a much longer non-coding region (6.9 kb) than the previously published NC_027673.1 (0.7 kb) and the non-coding regions of both genomes showed only partial homology, but with close homology of the intervening tRNA gene. We found the LNR to be comprised of two distinct repeat units with 8 and 13 copies, while the SNR was comprised of another distinct repeat unit with 3 copies (Fig. 2 and Additional File 1). Strikingly, five independent PacBio reads spanned the entirety of the non-coding region but with slight differences in length (6.3–6.9 kb), suggesting that the region is polymorphic, possibly even within individual worms.

### Repeat annotation

RepBase repeat consensus sequences did not adequately represent the repeats found in the *P. westermani* assembly, consistent with the distant evolutionary relationship of lung flukes with previously sequenced worm genomes. We therefore carried out *de novo* repeat characterization using the RepeatModeller package, version 1.0.9 (RepeatModeler, RRID:SCR_015027) and used the generated consensus sequences to identify repetitive regions by RepeatMasker (RepeatMasker, RRID:SCR_012954), version 4.0.7 (both available at [29]). To enable direct comparison with related trematode species, we also ran RepeatModeller and RepeatMasker separately on the *F. hepatica*, *O. viverrini*, and *C. sinensis* genomes with the same program parameters as those used for *P. westermani*.

A relatively high percentage (45.2%) of the *P. westermani* genome sequence was repeat derived, similar to the rate reported for *Schistosoma* spp. (40.1–47.5%) [20–22] and *F. hepatica* (57.1%) but considerably higher than the rate observed for the closer relatives *O. viverrini* (28.9%) and *C. sinensis* (32.6%) (Table 3). Retrotransposons of the long interspersed nuclear element (LINE) subtype were found to be the greatest contributors of repetitive DNA (21.6%) (Table 3), consistent with reports for other trematode genomes [23]. In *P. westermani* and *F. hepatica*, the two largest of the four included trematode genomes, long terminal repeat (LTR) retrotransposons were also highly abundant, contributing 7.7% and 10.1% of the genomes, respectively. Additionally, all four genomes had considerable amounts of repetitive DNA (10.7–17.1%) that did not match repeat consensus sequences of any of the known repeat classes modeled by RepeatModeler. The relatively large proportion of repeat-derived sequences in *P. westermani* may explain some of the increased size observed for this genome compared to the genomes of related flatworm species.

**Table 3:** Repeat content percentage of *P. westermani* and related trematode genome sequences

Repeat class	*P. westermani*	*F. hepatica*	*O. viverrini*	*C. sinensis*
LINE	21.57	26.17	12.76	14.85
LTR	7.71	10.06	2.82	1.97
DNA elements	1.76	2.14	0.94	1.04
SINE	0.96	1.06	1.26	1.22
Simple repeats	0.18	0.63	0.43	0.36
Unclassified	12.97	17.06	10.69	13.15
**Total**	**45.15**	**57.12**	**28.9**	**32.59**

### Gene prediction and functional annotation

Genes were predicted by the Maker pipeline, version 2.31.9, using Augustus [30], version 3.2.3, and GeneMark-ES [31], version 4.32, for *ab initio* gene prediction. To accurately model the sequence properties of the *P. westermani* genome, both gene finders were initially trained by BRAKER1 [32], version 1.9, which makes use of mapped transcriptome sequence data. Previously published RNA-sequencing (RNA-seq) data from adult *P. westermani* [9] were obtained from the short-read archive and mapped to our genome assembly using the Star aligner [33], version 2.5, with the option –twopassMode Basic. BRAKER1 was then run with default parameters. The RNA-seq data were further assembled into transcripts using cufflinks [34], version 2.2.1, with the options –frag-bias-correct <p.westermani assembly> –multi-read-correct. The resulting transcripts were provided as input for Maker via the ‘est_gff’ option. For homology-based searches Maker was provided with the following wormbase v8 protein datasets: *Clonorchis sinensis* (PRJDA72781), *Opisthorchis viverrini* (PRJNA222628), *Schistosoma mansoni* (PRJEA36577), *Caenorhabditis elegans* (PRJNA13758), *Echinococcus granulosus* (PRJEB121), *Hymenolepis diminuta* (PRJEB507), and *Schistosoma haematobium* (PRJNA78265). Additionally, the Swiss-Prot dataset from UniProt was included. Maker was allowed to report single exon genes and otherwise run with default parameters.

Proteins were functionally annotated based on a BLASTp search against the NCBI non-redundant protein database (obtained on 25.10.17) requiring an e-value <1e-15 and the best hit spanning at least 40% of the query sequence. Kyoto Encyclopedia of Genes and Genomes annotations were identified using the BlastKoala server with the option ‘genus_eukaryotes’ [35]. Additionally, functional domains, Gene Ontology (GO) annotations, transmembrane proteins, and signal peptides were identified with InterProScan (InterProScan, RRID:SCR_005829) [36], version 5.25–64.0. GO annotations were then visualized using WEGO [37]. In total, 12,852 protein encoding genes were predicted in the *P. westermani* genome and functionally annotated (Table 2).

### Genome comparison

**Figure 3:**
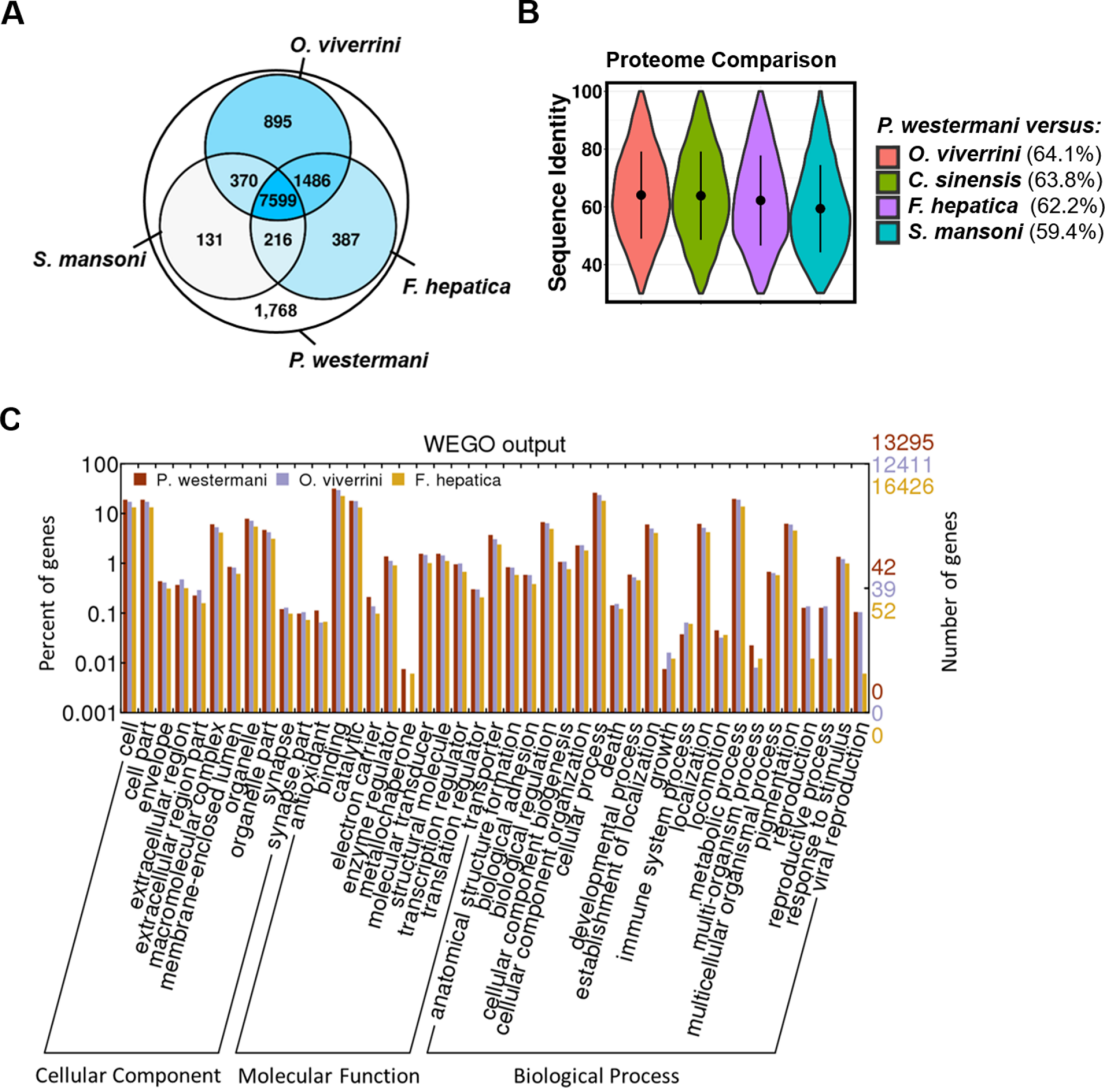
Conservation of the *P. westermani* proteome across four related trematode species. *Paragonimus westermani* proteins were mapped to the genome sequences of *O. viverrini, C. sinensis, F. hepatica*, and *S. mansoni* using Exonerate. **(A)***Paragonimus westermani* centered Venn diagram of 12,852 predicted proteins. The four included trematode species shared a core set of 7,599 proteins. **(B)** Sequence identity of *P. westermani* proteins and orthologues inferred in genomes of related trematodes. Average sequence identity is given in brackets. **(C)** Distribution of identified functional GO categories across three trematode species. GO annotations were assigned by InterProScan and visualized using WEGO.

Predicted *P. westermani* coding genes were mapped to the genomes of related trematode species using Exonerate, version 2.4.0, requiring a minimal sequence identity of 30% and excluding matches spanning less than 40% of the query protein. The majority of predicted proteins (86.2%) had inferred homologs in the related trematode species (Fig. 3A) and showed a similar distribution of protein functional categories (Fig. 3C). The *P. westermani*-predicted proteome was most similar to *O. viverrini* and *C. sinensis*. Of the 12,852 predicted proteins, 10,350 (80%) had inferred homologs in *O. viverrini* with an average sequence identity of 64.1%, and 10,227 (79.6%) had homologs in *C. sinensis* with an average sequence identity of 63.8% (Fig. 3A and 3B).

### Phylogenetic analysis and estimation of divergence time

**Figure 4:**
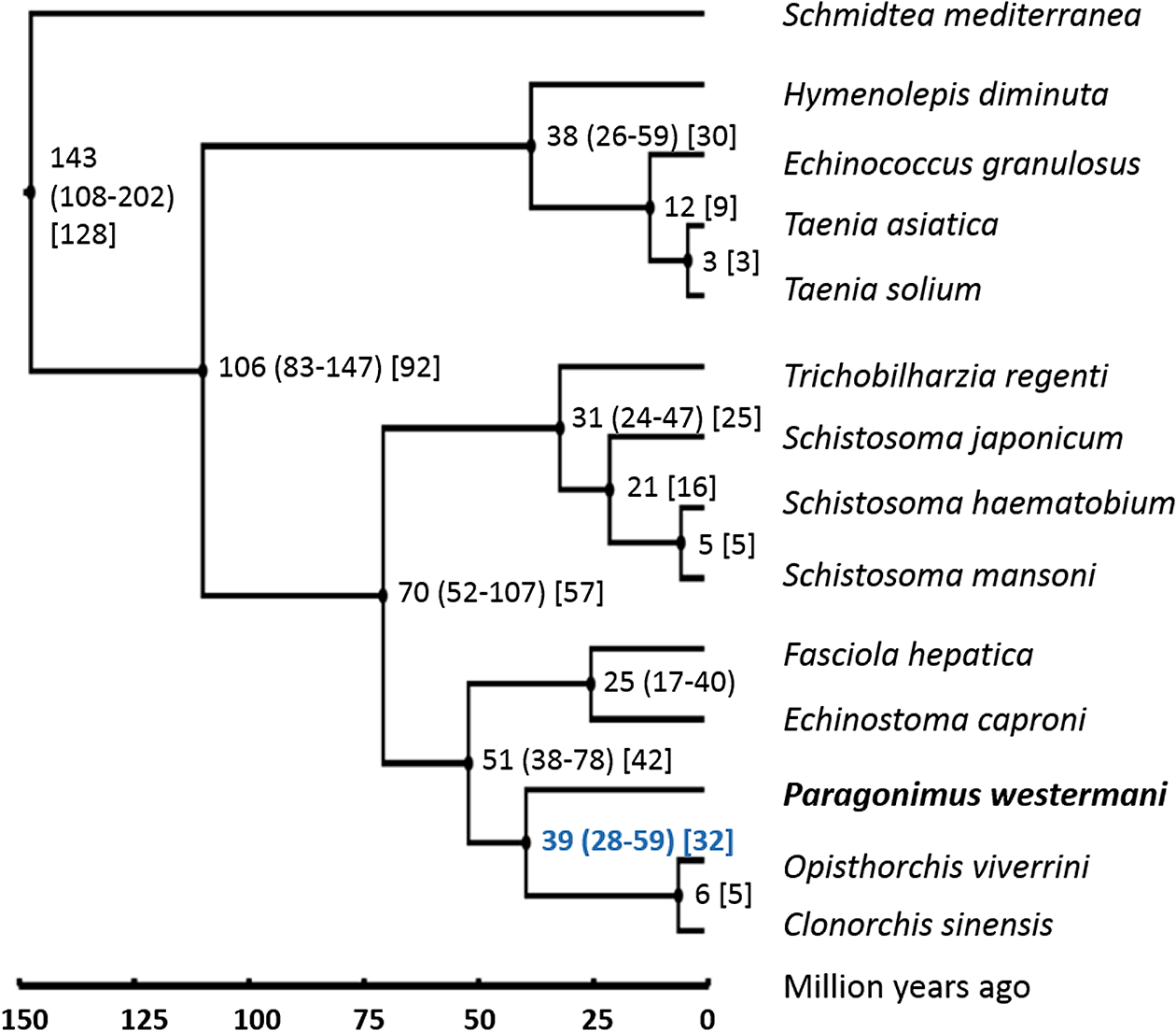
Phylogenetic tree and estimated divergence times. A phylogenetic tree of selected trematodes and cestodes and *S. mediterranea* as outgroup was reconstructed from 104 shared single-copy proteins using the maximum likelihood method. Species divergence was estimated by a Bayesian model using MCMCTREE with relaxed molecular clock and is given in million years, with 95% confidence intervals in round brackets. The split of *P. westermani* was estimated to have occurred somewhere around 38.9 million years ago (Mya; 28.0–58.6 million years). The analysis was repeated using BEAST 2, and estimated divergence times are shown in square brackets. BEAST 2 estimated the split of *P. westermani* to have occurred 31.5 Mya.

A protein-based phylogenetic tree was inferred from 14 worm genomes, including *P. westermani*, 12 related trematode/cestode species, and *Schmidtea mediterranea*, a free-living turbellarian flatworm, as outgroup (Fig. 4). We first identified single-copy proteins shared across all 14 included worm species. Single-copy proteins were identified based on BLASTp searches of a species proteins against the species own proteome using a sequence-identity cutoff of 30% and requiring hits to cover >50% of the query sequence. Single-copy proteins shared across all 14 species were then identified using a less stringent BLASTp search with a 30% sequence identity cutoff but requiring only >40% coverage of the query sequence. We identified 104 single-copy proteins shared across the 14 worm species that were then aligned using MUSCLE [38]. The resulting multiple sequence alignment was de-gapped with trimAI [39], and a phylogenetic tree was reconstructed by PhyML (PhyML, RRID:SCR_014629) [40]. Model selection in PhyML [41] identified the LG model [42] with decorations +G+I+F as optimal. PHYLIP v3.696 [43] using the maximum likelihood method and the Jones-Taylor-Thornton (JTT) probability model [44] resulted in the same tree topology, demonstrating the robustness of the inferred phylogenetic relationships.

The multiple alignment and the inferred phylogenetic tree were then used to estimate species divergence by a Bayesian model with relaxed molecular clock using MCMCTREE in PAML 4.9e (Fig. 4)(PAML, RRID:SCR_014932). The model was calibrated based on previously published divergence times and ages of fossil records. Evidence for trematode infestation have been reported from the Eocene (56 to 33.9 million years ago [Mya]) and preserved trematode eggs have been found in dinosaur coprolites from the Early Cretaceous (146 to 100 Mya); however, fossil records indicate that trematodes may have already existed more than 400 Mya [45, 46]. The trematode split from other neodermatan lineages was therefore fixed at >56 million years. The origin of schistosomes has been estimated somewhere in the Miocene around 15–20 Mya [47, 48]. It has further been estimated that the divergence of *S. mansoni* did likely not occur before 2–5 Mya, based on fossil records of its intermediate host *Biomphalaria* [49]. From these data, the split of Plagiorchiida (including *P. westermani*) and Opistorchiida (including *O. viverrini* and *C. sinensis*) was estimated to have occurred 38.9 Mya (95% confidence interval of 28.0–58.6 million years) (Fig. 4). To estimate the robustness of the inferred divergence times, the analysis was repeated using BEAST 2 version 2.5.0 [50], based on the JTT substitution matrix, gamma category count of 4, estimated substitution rate, relaxed clock log normal model, and a chain length of 6M [51, 52]. A maximum clade credibility tree using median node heights was generated by the BEAST 2 treeannotator tool. Divergence times inferred by BEAST 2 matched well with the MCMCTREE results and were within the estimated confidence intervals (Fig. 4). The split of the Plagiorchiida and the Opistorchiida was estimated to have occurred 31.5 Mya.

## Discussion

We have presented the first whole-genome sequence of a *Paragonimus* spp. worm, providing a valuable resource to the field that will aid our understanding of this group of clinically important parasites. The genome was found to be unusually large for a worm. This is a feature that at least in part appears attributable to an expansion of retrotransposable elements, rather than genome duplication events.

The mitochondrial genome was also found to be very large, comprising 20.3 kb. Such a large size appears to be a common feature of worms and results from a long repetitive region of unknown function. However, while this region appears to be a feature of most flatworms, it is rarely sequenced in full due to the technical challenges of sequencing long tandemly repeated sequences.

*Paragonimus westermani* has been described as a species complex with considerable genetic differences across geographic regions [2]. The genome presented herein is of an Indian isolate, and it will be of considerable interest to compare this and the genomes of isolates from other regions where *P. westermani* is endemic in order to elucidate the region-specific genetic features. This would be particularly informative as not all endemic regions are associated with paragonimiasis in humans [2].

Phylogenetic analyses of *P. westermani* shows that it has diverged considerably from its closest relatives, *Clonorchis sinensis* and *Opisthorchis viverrini*, with a split estimated to have occurred 28–59 Mya. Subsequent to that split, the species spread out across a vast geographical range, acquiring distinct local traits in what may eventually be considered speciation events. This time span has also seen an expansion of two repeat families, in particular, the LINE and LTR elements. In mammals, these elements are known to occasionally become exapted and gain novel regulatory functions [53], and they are therefore likely to add to the diversity of the *P. westermani* species complex.

## Conclusions

The presented *P. westermani* genome assembly provides new insights into the molecular biology of *Paragonimus* and provides an unprecedented resource for functional studies of lung flukes and for the design of new disease interventions and diagnostics tests.

## Availability of supporting data

The nuclear and mitochondrial genomes are available from NCBI under accession number PRJNA454344. Annotation and tree data is available from the *GigaScience* GigaDB repository [54].

## Additional file

Additional file 1.pdf

## Abbreviations

BLAST: Basic Local Alignment Search Tool; BUSCO: Benchmarking Universal Single-Copy Orthologs; GO: Gene Ontology; LINE: long interspersed nuclear element; LNR: long noncoding region; LTR: long terminal repeat; Mya: million years ago; NCBI: National Center for Biotechnology Information; PacBio: Pacifc Biosciences; PCR: polymerase chain reaction; RNA-seq: RNA sequencing; SNR: short non-coding region.

## Competing interests

All authors declare that they have no competing interests.

## Funding

This work has been supported by grants from the QIMR Berghofer Medical Research Institute (Chenhall Estate) and the Australian Infectious Diseases Research Centre. D.P.M.A is a National Health and Medical Research Council Senior Principal Research Fellow.

## Author contributions

L.K. and D.P.M. conceived and managed the project. K.N. and K.R.D. provided *P. westermani* material. T.A. and S.N. isolated genomic DNA. M.Z. and G.G. managed DNA sequencing. H.O. carried out genome assembly, gene prediction, and functional genome annotation. H.O. and L.K. carried out comparative genomics. L.K., D.P.M., M.K.J., and M.A.R. obtained funding and designed the study. L.K. and H.O. drafted the manuscript. All authors read, edited, and approved the final manuscript.

## Supplementary Material

GIGA-D-18-00193_Original_Submission.pdf

GIGA-D-18-00193_Revision_1.pdf

Response_to_Reviewer_Comments_Original_Submission.pdf

Reviewer_1_Report_(Original_Submission) -- Rodrigo Baptista, Ph.D. 7/11/2018 Reviewed

Reviewer_1_Report_(Revision_1) -- Rodrigo Baptista, Ph.D. 10/13/2018 Reviewed

Reviewer_2_Report_(Original_Submission) -- Krystyna Cwiklinski 7/16/2018 Reviewed

Supplemental File
