# Supplementary material for: Whole-genome sequence of the oriental lung fluke Paragonimus westermani
Source: Gigascience. 2018 Dec 6;8(1):giy146. doi: 10.1093/gigascience/giy146 (PMC6329441; doi:10.1093/gigascience/giy146)
Supplement: GIGA-D-18-00193_Original_Submission.pdf [file giy146_giga-d-18-00193_original_submission.pdf]

GigaScience

Complete genome sequence of the oriental lung fluke Paragonimus westermani

--Manuscript Draft--

**Manuscript Number:**	GIGA-D-18-00193
**Full Title:**	Complete genome sequence of the oriental lung fluke Paragonimus westermani
**Article Type:**	Research
**Funding Information:**	the QIMR Berghofer Medical Research Institute (Chenhall Estate) — A/Prof Lutz Krause<br>Australian Infectious Diseases Research Centre — A/Prof Lutz Krause<br>National Health and Medical Research Council — Prof Donald P. McManus
**Abstract:**	Background<br>Foodborne infections caused by lung flukes of the genus Paragonimus are a significant and widespread public health problem in tropical areas. Around 50 Paragonimus species have been reported to infect animals and humans, but Paragonimus westermani is responsible for the bulk of human disease. Despite their medical and economic importance, no genome sequence for any Paragonimus species is available.<br>Results<br>We sequenced and assembled the genome of P. westermani, which is among the largest of the known pathogen genomes with an estimated size of 1.1 Gb. A 924.5 Mb genome assembly was generated from Illumina and PacBio sequence data. The genome has a high proportion (45%) of repeat-derived DNA, particularly of the LINE and LTR subtypes, and the expansion of these elements may explain some of the large size. We predicted 12,852 protein coding genes, showing a high level of conservation with related trematode species. The majority of proteins (80%) had homologs in the human liver fluke Opisthorchis viverrini with an average sequence identity of 64.1%. Assembly of the P. westermani mitochondrial genome from long PacBio reads resulted in a single high-quality circularized 20.6 kb contig. The contig harboured a 6.9 kb region of non-coding repetitive DNA comprised of three distinct repeat units. Our results suggest that the region is highly polymorphic in P. westermani, possibly even within single worm isolates.<br>Conclusions<br>The generated assembly represents the first Paragonimus genome sequence and will facilitate future molecular studies of this important, but neglected, parasite group.
**Corresponding Author:**	Lutz Krause<br><br>AUSTRALIA
**Corresponding Author Secondary Information:**	
**Corresponding Author's Institution:**	
**Corresponding Author's Secondary Institution:**	
**First Author:**	Harald Oey
**First Author Secondary Information:**	
**Order of Authors:**	Harald Oey
	Martha Zakrzewski
	Kanwar Narain
	K. Rekha Devi
	Takeshi Agatsuma
	Sujeevi Nawaratna

Powered by Editorial Manager® and ProduXion Manager® from Aries Systems Corporation

	Geoffrey Gobart
	Malcolm Jones
	Mark A. Ragan
	Donald P. McManus
	Lutz Krause
**Order of Authors Secondary Information:**	
**Additional Information:**	
**Question**	**Response**
Are you submitting this manuscript to a special series or article collection?	No
**Experimental design and statistics**<br><br>Full details of the experimental design and statistical methods used should be given in the Methods section, as detailed in our Minimum Standards Reporting Checklist. Information essential to interpreting the data presented should be made available in the figure legends.<br><br>Have you included all the information requested in your manuscript?	Yes
**Resources**<br><br>A description of all resources used, including antibodies, cell lines, animals and software tools, with enough information to allow them to be uniquely identified, should be included in the Methods section. Authors are strongly encouraged to cite Research Resource Identifiers (RRIDs) for antibodies, model organisms and tools, where possible.<br><br>Have you included the information requested as detailed in our Minimum Standards Reporting Checklist?	Yes
**Availability of data and materials**<br><br>All datasets and code on which the conclusions of the paper rely must be either included in your submission or deposited in publicly available repositories (where available and ethically appropriate), referencing such data using a unique identifier in the references and in the "Availability of Data and Materials" section of your manuscript.	Yes

Powered by Editorial Manager® and ProduXion Manager® from Aries Systems Corporation

Have you have met the above requirement as detailed in our Minimum Standards Reporting Checklist?	

Powered by Editorial Manager® and ProduXion Manager® from Aries Systems Corporation

Manuscript
Click here to download Manuscript Paragonimus_GigaSciences_flat.docx

Title Page

Complete genome sequence of the oriental lung fluke *Paragonimus westermani*

Harald Oey[1, #], Martha Zakrzewski[2], Kanwar Narain[6], K. Rekha Devi[6], Takeshi Agatsuma[7], Sujeevi Nawaratna[2], Geoffrey Gobart[2,4], Malcolm Jones[3], Mark A. Ragan[5], Donald P. McManus[2,*], Lutz Krause[1,2,*,#]

[1] The University of Queensland Diamantina Institute, Brisbane, QLD, Australia

[2] QIMR Berghofer Medical Research Institute, Brisbane, QLD, Australia

[3] School of Veterinary Science, University of Queensland, Gatton, QLD, Australia

[4] School of Biological Sciences, Queen's University Belfast, UK

[5] Institute for Molecular Bioscience, The University of Queensland, Brisbane, QLD, Australia

[6] Regional Medical Research Centre for Northeastern region, Indian Council of Medical Research Department of Health Research Ministry of H & FW, Govt. of India, Dibrugarh-786 001, India

[7] Department of Environmental Medicine, Kochi Medical School, Kochi University, Kohasu, Oko, Nankoku City Kochi 783-8505

[*] authors contributed equally

[#] To whom correspondence should be addressed

Contact: l.krause@uq.edu.au and h.oey@uq.edu.au

Author names, affiliations and emails:

Dr Harald Oey

The University of Queensland Diamantina Institute, The Faculty of Medicine, The University of Queensland, Translational Research Institute, Brisbane, QLD

Email: h.oey@uq.edu.au

Dr Martha Zakrzewski

QIMR Berghofer Medical Research Institute, Brisbane, QLD, Australia

Email: Martha.Zakrzewski@qimrberghofer.edu.au

Dr Kanwar Narain

Regional Medical Research Centre for Northeastern region, Indian Council of Medical Research Department of Health Research Ministry of H & FW, Govt. of India, Dibrugarh-786 001, India

Email: kanwar_narain@hotmail.com

Dr K. Rekha Devi

Regional Medical Research Centre for Northeastern region, Indian Council of Medical Research Department of Health Research Ministry of H & FW, Govt. of India, Dibrugarh-786 001, India

Email: krekha75@yahoo.co.in

Prof Takeshi Agatsuma

Department of Environmental Medicine

Kochi Medical School, Kochi University, Kohasu, Oko, Nankoku City Kochi 783-8505

Email: agatsuma@kochi-u.ac.jp

Dr Geoffrey Gobert

School of Biological Sciences, Queen’s University Belfast, UK

Email: g.gobert@qub.ac.uk

Prof Mark A. Ragan

Institute for Molecular Bioscience, The University of Queensland, Brisbane, QLD, Australia

Email: m.ragan@uq.edu.au

Prof Malcolm Jones

School of Veterinary Science, University of Queensland, Gatton, QLD, Australia

Email: m.jones@uq.edu.au

Dr Sujeevi Nawaratna

QIMR Berghofer Medical Research Institute, Brisbane, QLD, Australia

Email: sujeevi.nawaratna@qimrberghofer.edu.au

Prof Donald P. McManus

QIMR Berghofer Medical Research Institute, Brisbane, QLD, Australia

Email: Don.McManus@qimrberghofer.edu.au

A/Prof Lutz Krause

The University of Queensland Diamantina Institute, The Faculty of Medicine, The University of Queensland, Translational Research Institute, Brisbane, QLD

Email: L.Krause@uq.edu.au

Abstract

Background

Foodborne infections caused by lung flukes of the genus *Paragonimus* are a significant and widespread public health problem in tropical areas. Around 50 *Paragonimus* species have been reported to infect animals and humans, but *Paragonimus westermani* is responsible for the bulk of human disease. Despite their medical and economic importance, no genome sequence for any *Paragonimus* species is available.

Results

We sequenced and assembled the genome of *P. westermani*, which is among the largest of the known pathogen genomes with an estimated size of 1.1 Gb. A 924.5 Mb genome assembly was generated from Illumina and PacBio sequence data. The genome has a high proportion (45%) of repeat-derived DNA, particularly of the LINE and LTR subtypes, and the expansion of these elements may explain some of the large size. We predicted 12,852 protein coding genes, showing a high level of conservation with related trematode species. The majority of proteins (80%) had homologs in the human liver fluke *Opisthorchis viverrini* with an average sequence identity of 64.1%. Assembly of the *P. westermani* mitochondrial genome from long PacBio reads resulted in a single high-quality circularized 20.6 kb contig. The contig harboured a 6.9 kb region of non-coding repetitive DNA comprised of three distinct repeat units. Our results suggest that the region is highly polymorphic in *P. westermani*, possibly even within single worm isolates.

Conclusions

The generated assembly represents the first *Paragonimus* genome sequence and will facilitate future molecular studies of this important, but neglected, parasite group.

Keywords

Paragonimus westermani, whole-genome sequence, genome assembly, paragonimiasis, food-borne disease, oriental lung fluke, parasitic infection, bioinformatics, high-throughput sequencing, comparative genomics, genome annotation, neglected tropical disease, flatworm

Background

Paragonimus lung flukes represent a significant and widespread clinical problem with an estimated 23 million people infected worldwide [1]. Around 50 species are described, with at least 7 being human pathogens [2]. The majority of human *Paragonimus* infections can be attributed to the *P. westermani* species complex, mainly in Southeast Asia and Japan [1]. *P. westermani* show considerable geographic genetic variability and human infections occur predominantly in East Asia and the Philippines. In India the incidence rates of paragonimiasis caused by *P. westermani* is currently unknown [2-4], however many cases of paragonimiasis are attributed to the related worm *Paragonimus heterotremus* [2]. Paragonimiasis is a zoonotic disease and also pigs, dogs and other animals can harbour *P. westermani* [2].

Paragonimus spp. have a complex life cycle. Unembryonated eggs are expelled by coughing or passed with faeces and develop in water. Miracidia hatch from the eggs and penetrate a freshwater snail, its first intermediate host. During several asexual developmental phases inside the snail, miracidia develop into a sporocysts and then two redial generations occur, the second of which giving rise to microcercous cercariae that escape into fresh water. These crawling cercariae invade a species of crustacean, the second intermediate host, to encyst in muscles and other sites and develop into metacercariae. Humans and other definitive hosts become infected through consumption of raw or inadequately cooked freshwater crabs or crayfish [5]. Ingested metacercariae excyst, penetrate through the gut and become encapsulated in the lungs where they mature into hermaphroditic adult worms (7.5 mm to 12 mm in length) in 6-10 weeks [5]. Paragonimiasis can lead to a chronic inflammatory disease of the lung and can trigger asthma- or tuberculosis-like symptoms [6-8]. In more severe cases *Paragonimus* can infect the brain or central nervous system of the definitive host, leading to headache, visual loss, and even death [1].

Paragonimiasis is commonly diagnosed by microscopic detection of parasite eggs in faeces or sputum. The lack of sensitive and reliable diagnostic tests in conjunction with unspecific disease symptoms often leads to delayed treatment with the drug of choice, praziquantel [8]. Despite their high medical, veterinary and economic importance, only limited information on the molecular biology of *Paragonimus* is currently available. Recent transcriptome sequencing studies have provided some information on the gene content of *Paragonimus* [9],

but until now no *Paragonimus* genome sequence has been available. Here we present a 924.5 Mb assembly of the *P. westermani* genome which provides new insights into the genomic composition of the *Paragonimus* genus and represents an invaluable resource for future studies of the neglected tropical disease paragonimiasis.

Data Description

Sequencing

Diploid *Paragonimus westermani* metacercariae were collected from the freshwater crab *Maydelliathelphusa lugubris* in 2009 in the East Siang district of Arunachal Pradesh, Northeast India, and fed to Wistar rats as experimental hosts. Genomic DNA was isolated from 50 individual worms, yielding 18 µg of DNA, and 2 µg of DNA was used to sequence the *Paragonimus westermani* genome using a whole-genome shotgun approach. Paired-end short-insert (200 bp and 450 bp) and mate-pair (5 Kb and 10 Kb) genomic DNA libraries were sequenced on the Illumina HiSeq platform, yielding 58 Gb of sequence data (**Table 1**). For genome scaffolding and quality evaluation of the assembled sequence, additional long-read data were generated using the PacBio platform, yielding 1.7 Gb of information (**Table 1**). The genome size was estimated from the K-mer coverage of the 450 bp insert library. K-mer frequencies were calculated by the program Jellyfish[10], version 2.2.6 , using a K-mer size of 17bp. The 17-mer distribution in the 450 bp library had a single peak at 26x (**Figure 1**), demonstrating low sequence heterozygosity. The genome size (G) was deduced from the K-mer distribution via the formula $G = N * (L – K + 1) / K\_depth$[11], where N is the total number of reads, L is the read length, K is the K-mer size and K_depth is the peak frequency. With an estimated size of 1.1 Gb the *P. westermani* genome is among the largest known pathogen genomes and one of the largest parasite genomes sequenced to date. The genome is considerably larger than the published genomes of the related trematodes *Clonorchis sinensis* (644 Mb)[12] , *Opisthorchis viverrini* (assembly size of 634.5 Mb) [13], and *Schistosoma* spp (381-403 Mb) [14-16] and comparable to the 1.3 Gp genome of *Fasciola hepatica* [17].

Table 1. ***Paragonimus westermani*** **sequencing libraries.**

Library	Platform	Library type	Insert size (bp)	Read length (bp)	Read count (raw)
200bp	HiSeq	Paired-end	200	2 x 120	140,542,299
450bp	HiSeq	Paired-end	450	2 x 100	171,954,230
5kb	HiSeq	Mate-pair	5,000	2 x 49	232,630,904
10kb	HiSeq	Mate-pair	10,000	2 x 49	266,480,540
PacBio	PacBio	Long read	-	-	1,731,327

Genome assembly

PacBio sequence data were error corrected by proovread version 2.13.13 [18], using Illumina short reads from the 200bp and 450bp libraries as input , and assembled into contigs by Mira v4.0.2 [19]. Short-read Illumina sequence data were trimmed using Trimmomatic v0.36 and subsequently error corrected by KmerFreq_HA (part of SoapDenovo2 [20]) with a K-mer size of 23. The 10 kb mate-pair library showed a high proportion of PCR duplicates and was subjected to PCR de-duplication prior to genome assembly. Illumina paired-end sequence data were assembled using the ABYSS assembly pipeline [21], version 2.0.2, with options n=5 s=200 N=36 S=500 k=33 and including the PacBio contigs via the re-scaffolding feature. The resulting assembly was de-gapped using the SoapDenovo2 GapCloser program [20]. Mate-pair libraries were then used to scaffold the assembly with SSPACE v3.0[22] (with options -x 0 -a 0.60 -n 30 -z 200 -g 0) and gaps were again filled with GapCloser. To detect and resolve scaffolding errors, the resulting assembly was processed by the program REAPER [23] using the 5kb mate-pair library as input, breaking the assembly at sites with poor evidence for contiguity.

The assembly resulted in a 924.5 Mb genome sequence (30,997 scaffolds with N50 of 135 Kb) (**Table 2**), covering 84.0% of the estimated genome size. The GC content of the genome was 43.3%, comparable to genomes of other related trematodes (**Table 2**). Genome assembly completeness was evaluated by BUSCO [24] using the metazoan lineage data, resulting in similar scores to those obtained for the genomes of comparable trematode species (**Table 2**). The proportion of duplicated genes reported by BUSCO was also similar to that of comparable

trematodes suggesting that the relatively large size of the *P. westermani* genome is not the result of genome duplication events.

Table 2. Assembly statistics for *P. westermani* and comparable trematode genomes of similar size.

	P. westermani	***F. hepatica***	***O. viverrini***	***C. sinensis***
Assembly size (Mb)[a]	924.5	1,275.0	606.0	546.9
Total base pairs (Mb)[b]	877.7	1,183.5	558.0	547.1
Contig N50 (Kb)	7.0 (>100bp)	9.7	NA	14.7
Scaffold N50 (Kb)	135 (>1Kb)	204	1,324	30.2
Scaffold count	30,997 (>1Kb)	45,354 (>1kb)	4,919 (>1kb)	31,822
GC content (%)	43.3	44.1	43.8	44.1
Repeat content (%)	45.2	57.1	28.9	32.6
Protein coding genes	12,852	15,740[c]	16,356	13,634
Longest scaffold (Kb)	809	1,565	9,657	2,050
BUSCOS - Complete	65.3%	65.8%	71.4%	70.8%
BUSCOS - Duplicated	1.4%	0.8%	1.1%	1.5%
BUSCOS - Missing	25.8%	25.4%	23.0%	23.1%

[a]Combined length of all scaffolds. [b]Combined length of all scaffolds without gaps (N's). [c]Non-overlapping RNAseq-supported gene models[17]

Mitochondrial genome

The mitochondrial genome of *P. westermani* is present at a much higher copy number than the nuclear genome and we were able to assemble the full mitochondrial genome at high coverage from error corrected long PacBio reads using the Mira assembler[19], version 4.0.2. This resulted in a single mitochondrial contig of 20.3 Kb. The accuracy of the contig was confirmed by mapping short insert paired-end sequences directly onto the contig revealing single nucleotide discrepancies at only 4 positions. The mitochondrial genome was found to closely match previously published *Paragonimus* mitochondrial genomes with the best match from a blast search against the Nucleotide collection at the NCBI (https://blast.ncbi.nlm.nih.gov/Blast.cgi) being accession NC_027673.1, a *P. westermani*

complex sp. type 1 mitochondrial genome isolated in India (97% sequence identity across 13.4 Kb of NC_027673.1). This sequence was used as reference for mitochondrial gene identification and annotation, supplemented by mitochondrial gene predictions by Mitos [25]. The mitochondrial genomes of flatworms are known to harbour a region of non-coding repetitive DNA, generally comprised of a long noncoding region (LNR) and a short non-coding region (SNR) with a single tRNA gene separating them[26]. Reconstructing this region from short-read data proved challenging, but our long-read PacBio data allowed complete assembly of the repetitive region and circularization of the genome. Interestingly, our assembled mitochondrial genome sequence had a much longer non-coding region (6.9 Kb) than the previously published NC_027673.1 (0.7 Kb) and the non-coding regions of both genomes showed only partial homology, but with close homology of the intervening tRNA gene. We found the LNR to be comprised of two distinct repeat units with 8 and 13 copies while the SNR was comprised of another distinct repeat unit with 3 copies. Strikingly, five independent PacBio reads spanned the entirety of the non-coding region but with slight differences in length (6.3 – 6.9 Kb), suggesting that the region is polymorphic, possibly even within individual worms.

Repeat annotation

RepBase repeat consensus sequences did not adequately represent the repeats found in the *P. westermani* assembly, consistent with the distant evolutionary relationship of lung flukes with previously sequenced worm genomes. We therefore carried out *de-novo* repeat characterization using the RepeatModeller package, version 1.0.9, and used the generated consensus sequences to identify repetitive regions by RepeatMasker, version 4.0.7 (both available at http://www.repeatmasker.org). To enable direct comparison with related trematode species we also ran RepeatModeller and RepeatMasker separately on the *F. hepatica*, *O. viverrini* and *C. sinensis* genomes with the same program parameters as those used for *P. westermani*.

A relatively high percentage (45.2%) of the *P. westermani* genome sequence was repeat-derived, similar to the rate reported for *Schistosoma* spp. (40.1-47.5%) [14-16] and *F. hepatica* (57.1%), but considerably higher than the rate observed for the closer relatives *O. viverrini*

(28.9%) and *C. sinensis* (32.6%) (**Table 3**). Retrotransposons of the long interspersed nuclear element (LINE) subtype were found to be the greatest contributors of repetitive DNA (21.6%) (**Table 3**), consistent with reports for other trematode genomes [17]. In *P. westermani* and *F. hepatica*, the two largest of the four included trematode genomes, long terminal repeat (LTR) retrotransposons were also highly abundant contributing 7.7% and 10.1% of the genomes, respectively. Additionally, all four genomes had considerable amounts of repetitive DNA (10.7-17.1%) that did not match repeat consensus sequences of any of the known repeat classes modelled by RepeatModeler. The relatively high proportion of repeat-derived sequences in *P. westermani* may explain some of the increased size observed for this genome compared to the genomes of related flatworm species.

Table 3. Repeat content percentage of *P. westermani* and related trematode genome sequences.

Repeat class	*P. westermani*	*F. hepatica*	*O. viverrini*	*C. sinensis*
LINE	21.57	26.17	12.76	14.85
LTR	7.71	10.06	2.82	1.97
DNA elements	1.76	2.14	0.94	1.04
SINE	0.96	1.06	1.26	1.22
Simple repeats	0.18	0.63	0.43	0.36
Unclassified	12.97	17.06	10.69	13.15
**Total**	**45.15**	**57.12**	**28.9**	**32.59**

Gene prediction and functional annotation

Genes were predicted by the Maker pipeline, version 2.31.9, using Augustus [27], version 3.2.3, and GeneMark-ES [28], version 4.32, for *ab-initio* gene prediction. To accurately model the sequence properties of the *P. westermani* genome, both gene finders were initially trained by BRAKER1 [29], version 1.9, which makes use of mapped transcriptome sequence data. Previously published *P. westermani* RNA-seq data [9] were obtained from the short read archive and mapped to our genome assembly using the Star aligner [30], version 2.5, with the option --twopassMode Basic. BRAKER1 was then run with default parameters. The RNA-seq data was further assembled into transcripts using cufflinks [31], version 2.2.1, with the

options --frag-bias-correct <p.westermani assembly> --multi-read-correct. The resulting transcripts were provided as input for Maker via the "est_gff" option. For homology based searches Maker was provided with the following wormbase v8 protein datasets: *Clonorchis sinensis* (PRJDA72781), *Opisthorchis viverrini* (PRJNA222628), *Schistosoma mansoni* (PRJEA36577), *Caenorhabditis elegans* (PRJNA13758), *Echinococcus granulosus* (PRJEB121), *Hymenolepis diminuta* (PRJEB507) and *Schistosoma haematobium* (PRJNA78265). Additionally, the Swiss-Prot dataset from UniProt was included. Maker was allowed to report single exon genes, and otherwise run with default parameters.

Proteins were functionally annotated based on a BLASTp search against the NCBI non-redundant protein database (obtained on 25.10.17) requiring an e-value <1e-15 and the best hit spanning at least 40% of the query sequence. Additionally, functional domains, GO annotations, transmembrane proteins and signal peptides were identified with InterProScan [32], version 5.25-64.0. GO annotations were then visualized using WEGO [33]. In total, 12,852 protein encoding genes were predicted in the *P. westermani* genome and functionally annotated (**Table 2**).

Genome comparison

Predicted *P. westermani* coding genes were mapped to the genomes of related trematode species using Exonerate, version 2.4.0, requiring a minimal sequence identity of 30% and excluding matches spanning less than 40% of the query protein. The majority of predicted proteins (86.2%) had inferred homologs in the related trematode species (**Figure 3A**) and showed a similar distribution of protein functional categories (**Figure 3C**). The *P. westermani* proteome was most similar to *O. viverrini* and *C. sinensis*. Of the 12,852 predicted proteins, 10,350 (80%) had inferred homologs in *O. viverrini* with an average sequence identity of 64.1%, and 10,227 (79.6%) had homologs in *C. sinensis* with an average sequence identity of 63.8% (**Figures 3A and 3B**).

Phylogenetic analysis and estimation of divergence time

A protein-based phylogenetic tree was inferred from 14 worm genomes, including *P. westermani,* 12 related trematode/cestode species and *Schmidtea mediterranea,* a free-

living turbellarian flatworm, as outgroup (**Figure 4**). We first identified single-copy proteins shared across all 14 included worm species. Single-copy proteins were identified based on blastp searches of a species proteins against the species own proteome using a sequence-identity cut-off of 30% and requiring hits to cover >50% of the query sequence. Single-copy proteins shared across all 14 species were then identified using a less stringent blastp search with a 30% sequence identity cut-off but requiring only >40% coverage of the query sequence. We identified 104 single-copy proteins shared across the 14 worm species that were then aligned using MUSCLE [34]. The resulting multiple sequence alignment was de-gapped with trimAI [35] and a phylogenetic tree was reconstructed by PHYLIP v3.696 using the maximum likelihood method and the Jones-Taylor-Thornton probability model.

The multiple alignment and the inferred phylogenetic tree were then used to estimate species divergence by a Bayesian model with relaxed molecular clock using MCMCTREE in PAML 4.9e (**Figure 4**). The model was calibrated based on previously published divergence times and ages of fossil records. Evidence for trematode infestation have been reported from the Eocene (56 to 33.9 million years (myr) ago) and preserved trematode eggs have been found in dinosaur coprolites from the Early Cretaceous (146 to 100 myr ago); however, fossil records indicate that trematodes may have already existed more than 400 myr ago [36, 37]. The trematode split from other neodermatan lineages was therefore fixed at >56 myr. The origin of schistosomes has been estimated somewhere in the Miocene around 15-20 myr ago [38, 39]. It has further been estimated that the divergence of *S. mansoni* did likely not occur before 2-5 myr ago, based on fossil records of its intermediate host *Biomphalaria* [40]. From these data, the split of Plagiorchiida (including *P. westermani*) and Opistorchiida (including *O. viverrini* and *C. sinensis*) was estimated to have occurred 38.9 myr ago (95% confidence interval of 28.0-58.6 myr) (**Figure 4**).

Conclusion

The presented *P. westermani* genome assembly provides new insights into the molecular biology of *Paragonimus* and provides an unprecedented resource for functional studies of lung flukes and for the design of new disease interventions and diagnostics tests.

Availability of supporting data

The nuclear and mitochondrial genomes are available from NCBI under accession number PRJNA454344

Abbreviations

LTR	Long terminal repeat
SINE	Short interspersed nuclear elements
LINE	Long interspersed nuclear elements
MYR	Million years

Competing interests

All authors declare that they have no competing interests.

Funding

This work has been supported by grants from the QIMR Berghofer Medical Research Institute (Chenhall Estate) and the Australian Infectious Diseases Research Centre. DPM is a National Health and Medical Research Council Senior Principal Research Fellow.

Author contributions

LK and DPM conceived and managed the project; KN and KRD provided *P. westermani* material. TA and SN isolated genomic DNA. MZ and GG managed DNA sequencing. HO carried out genome assembly, gene prediction and functional genome annotation. HO and LK carried out comparative genomics. LK, DPM, MKJ and MAR attracted funding and designed the study.

LK and HO drafted the manuscript and all authors read, edited and approved the final manuscript.

Acknowledgements

We wish to acknowledge the QIMR Berghofer Medical Research Institute (Chenhall Estate donation) and the Australian Infectious Diseases Research Centre for funding this work.

References

1. Furst T, Keiser J and Utzinger J. Global burden of human food-borne trematodiasis: a systematic review and meta-analysis. Lancet Infect Dis. 2012;12 3:210-21. doi:10.1016/S1473-3099(11)70294-8.
2. Blair D. Paragonimiasis. Adv Exp Med Biol. 2014;766:115-52. doi:10.1007/978-1-4939-0915-5_5.
3. Roy JS, Das PP, Borah AK and Das JK. Paragonimiasis in a Child from Assam, India. J Clin Diagn Res. 2016;10 4:DD06-7. doi:10.7860/JCDR/2016/18160.7616.
4. Singh TS, Hiromu S, Devi KR and Singh WA. First case of Paragonimus westermani infection in a female patient in India. Indian J Med Microbiol. 2015;33 Suppl:156-9. doi:10.4103/0255-0857.150950.
5. Jones MK, Keiser J and McManus DP. Trematodes. In: Jorgensen JH, Pfaller MA, Carroll KC, Funke G, Landry ML, Richter SS, et al., editors. Manual of Clinical Microbiology, Eleventh Edition. American Society of Microbiology; 2015.
6. Luo J, Wang MY, Liu D, Zhu H, Yang S, Liang BM, et al. Pulmonary Paragonimiasis Mimicking Tuberculous Pleuritis: A Case Report. Medicine (Baltimore). 2016;95 15:e3436. doi:10.1097/MD.0000000000003436.
7. Zhou R, Zhang M, Cheng N and Zhou Y. Paragonimiasis mimicking chest cancer and abdominal wall metastaisis: A case report. Oncol Lett. 2016;11 6:3769-71. doi:10.3892/ol.2016.4434.
8. Kalhan S, Sharma P, Sharma S, Kakria N, Dudani S and Gupta A. Paragonimus westermani infection in lung: A confounding diagnostic entity. Lung India. 2015;32 3:265-7. doi:10.4103/0970-2113.156248.
9. Li BW, McNulty SN, Rosa BA, Tyagi R, Zeng QR, Gu KZ, et al. Conservation and diversification of the transcriptomes of adult Paragonimus westermani and P. skrjabini. Parasit Vectors. 2016;9:497. doi:10.1186/s13071-016-1785-x.
10. Marcais G and Kingsford C. A fast, lock-free approach for efficient parallel counting of occurrences of k-mers. Bioinformatics. 2011;27 6:764-70. doi:10.1093/bioinformatics/btr011.
11. Song L, Bian C, Luo Y, Wang L, You X, Li J, et al. Draft genome of the Chinese mitten crab, Eriocheir sinensis. Gigascience. 2016;5:5. doi:10.1186/s13742-016-0112-y.
12. Wang X, Chen W, Huang Y, Sun J, Men J, Liu H, et al. The draft genome of the carcinogenic human liver fluke Clonorchis sinensis. Genome Biol. 2011;12 10:R107. doi:10.1186/gb-2011-12-10-r107.

13. Young ND, Nagarajan N, Lin SJ, Korhonen PK, Jex AR, Hall RS, et al. The Opisthorchis viverrini genome provides insights into life in the bile duct. Nat Commun. 2014;5:4378. doi:10.1038/ncomms5378.
14. Young ND, Jex AR, Li B, Liu S, Yang L, Xiong Z, et al. Whole-genome sequence of Schistosoma haematobium. Nat Genet. 2012;44 2:221-5. doi:10.1038/ng.1065.
15. Berriman M, Haas BJ, LoVerde PT, Wilson RA, Dillon GP, Cerqueira GC, et al. The genome of the blood fluke Schistosoma mansoni. Nature. 2009;460 7253:352-8. doi:10.1038/nature08160.
16. Schistosoma japonicum Genome S and Functional Analysis C. The Schistosoma japonicum genome reveals features of host-parasite interplay. Nature. 2009;460 7253:345-51. doi:10.1038/nature08140.
17. Cwiklinski K, Dalton JP, Dufresne PJ, La Course J, Williams DJ, Hodgkinson J, et al. The Fasciola hepatica genome: gene duplication and polymorphism reveals adaptation to the host environment and the capacity for rapid evolution. Genome Biol. 2015;16:71. doi:10.1186/s13059-015-0632-2.
18. Hackl T, Hedrich R, Schultz J and Forster F. proovread: large-scale high-accuracy PacBio correction through iterative short read consensus. Bioinformatics. 2014;30 21:3004-11. doi:10.1093/bioinformatics/btu392.
19. Chevreux B, Pfisterer T, Drescher B, Driesel AJ, Muller WE, Wetter T, et al. Using the miraEST assembler for reliable and automated mRNA transcript assembly and SNP detection in sequenced ESTs. Genome research. 2004;14 6:1147-59. doi:10.1101/gr.1917404.
20. Luo R, Liu B, Xie Y, Li Z, Huang W, Yuan J, et al. SOAPdenovo2: an empirically improved memory-efficient short-read de novo assembler. Gigascience. 2012;1 1:18. doi:10.1186/2047-217X-1-18.
21. Simpson JT, Wong K, Jackman SD, Schein JE, Jones SJ and Birol I. ABySS: a parallel assembler for short read sequence data. Genome research. 2009;19 6:1117-23. doi:10.1101/gr.089532.108.
22. Boetzer M, Henkel CV, Jansen HJ, Butler D and Pirovano W. Scaffolding pre-assembled contigs using SSPACE. Bioinformatics. 2011;27 4:578-9. doi:10.1093/bioinformatics/btq683.
23. Hunt M, Kikuchi T, Sanders M, Newbold C, Berriman M and Otto TD. REAPR: a universal tool for genome assembly evaluation. Genome Biol. 2013;14 5:R47. doi:10.1186/gb-2013-14-5-r47.
24. Simao FA, Waterhouse RM, Ioannidis P, Kriventseva EV and Zdobnov EM. BUSCO: assessing genome assembly and annotation completeness with single-copy orthologs. Bioinformatics. 2015;31 19:3210-2. doi:10.1093/bioinformatics/btv351.
25. Bernt M, Donath A, Juhling F, Externbrink F, Florentz C, Fritzsch G, et al. MITOS: improved de novo metazoan mitochondrial genome annotation. Mol Phylogenet Evol. 2013;69 2:313-9. doi:10.1016/j.ympev.2012.08.023.
26. Le TH, Blair D and McManus DP. Mitochondrial genomes of parasitic flatworms. Trends Parasitol. 2002;18 5:206-13.
27. Stanke M, Steinkamp R, Waack S and Morgenstern B. AUGUSTUS: a web server for gene finding in eukaryotes. Nucleic Acids Res. 2004;32 Web Server issue:W309-12. doi:10.1093/nar/gkh379.
28. Lomsadze A, Ter-Hovhannisyan V, Chernoff YO and Borodovsky M. Gene identification in novel eukaryotic genomes by self-training algorithm. Nucleic Acids Res. 2005;33 20:6494-506. doi:10.1093/nar/gki937.
29. Hoff KJ, Lange S, Lomsadze A, Borodovsky M and Stanke M. BRAKER1: Unsupervised RNA-Seq-Based Genome Annotation with GeneMark-ET and AUGUSTUS. Bioinformatics. 2016;32 5:767-9. doi:10.1093/bioinformatics/btv661.
30. Dobin A, Davis CA, Schlesinger F, Drenkow J, Zaleski C, Jha S, et al. STAR: ultrafast universal RNA-seq aligner. Bioinformatics. 2013;29 1:15-21. doi:10.1093/bioinformatics/bts635.

31. Trapnell C, Williams BA, Pertea G, Mortazavi A, Kwan G, van Baren MJ, et al. Transcript assembly and quantification by RNA-Seq reveals unannotated transcripts and isoform switching during cell differentiation. Nat Biotechnol. 2010;28 5:511-5. doi:10.1038/nbt.1621.
32. Jones P, Binns D, Chang HY, Fraser M, Li W, McAnulla C, et al. InterProScan 5: genome-scale protein function classification. Bioinformatics. 2014;30 9:1236-40. doi:10.1093/bioinformatics/btu031.
33. Ye J, Fang L, Zheng H, Zhang Y, Chen J, Zhang Z, et al. WEGO: a web tool for plotting GO annotations. Nucleic Acids Res. 2006;34 Web Server issue:W293-7. doi:10.1093/nar/gkl031.
34. Edgar RC. MUSCLE: multiple sequence alignment with high accuracy and high throughput. Nucleic Acids Res. 2004;32 5:1792-7. doi:10.1093/nar/gkh340.
35. Capella-Gutierrez S, Silla-Martinez JM and Gabaldon T. trimAl: a tool for automated alignment trimming in large-scale phylogenetic analyses. Bioinformatics. 2009;25 15:1972-3. doi:10.1093/bioinformatics/btp348.
36. Poinar G, Jr. and Boucot AJ. Evidence of intestinal parasites of dinosaurs. Parasitology. 2006;133 Pt 2:245-9. doi:10.1017/S0031182006000138.
37. Huntley JW and De Baets K. Trace Fossil Evidence of Trematode-Bivalve Parasite-Host Interactions in Deep Time. Adv Parasitol. 2015;90:201-31. doi:10.1016/bs.apar.2015.05.004.
38. Littlewood DTJe and Baets Kde. Fossil parasites.
39. Snyder SD and Loker ES. Evolutionary relationships among the Schistosomatidae (Platyhelminthes:Digenea) and an Asian origin for Schistosoma. J Parasitol. 2000;86 2:283-8. doi:10.1645/0022-3395(2000)086[0283:ERATSP]2.0.CO;2.
40. Morgan JA, Dejong RJ, Snyder SD, Mkoji GM and Loker ES. Schistosoma mansoni and Biomphalaria: past history and future trends. Parasitology. 2001;123 Suppl:S211-28.

Figure legends:

Figure 1. K-mer frequencies for the 450bp library. Distribution of 17-mers in the 450bp short-insert library demonstrated low sequence heterozygosity. We observed a single peak at 26x and the *P. westermani* genome size was estimated to be 1.1 Gb.

Figure 2. The complete *P. westermani* mitochondrial genome.

A) A graphical representation of the *P. westermani* circular mitochondrial genome is shown, including a ~6.9 Kb repetitive region. Three distinct repeat units were identified in this region, as well as an intervening tRNA gene (tRNA-Glu). All genes are transcribed in the clock-wise direction. B) The consensus sequence for three repeat units identified in the ~6.9 Kb repetitive region.

Figure 3. Conservation of the *P. westermani* proteome across four related trematode species. *P. westermani* proteins were mapped to the genome sequences of *O. viverrini, C. sinensis, F. hepatica* and *S. mansoni* using Exonerate. A) *P. westermani* centred Venn diagram of 12,852 predicted proteins. The four included trematode species shared a core set of 7,599 proteins. B) Sequence identity of *P. westermani* proteins and orthologues inferred in genomes of related trematodes. Average sequence identity is given in brackets. C) Distribution of identified functional GO categories across three trematode species. GO annotations were assigned by InterProScan and visualized using WEGO.

Figure 4. Phylogenetic tree and estimated divergence times. A phylogenetic tree of selected trematodes and cestodes and *S. mediterranea* as outgroup was reconstructed from 104 shared single-copy proteins using the maximum likelihood method. Species divergence was estimated by a Bayesian model with relaxed molecular clock and is given in million years with 95% confidence intervals in brackets. The split of *P. westermani* was estimated to have occurred somewhere around 38.9 myr ago (28.0-58.6 myr).

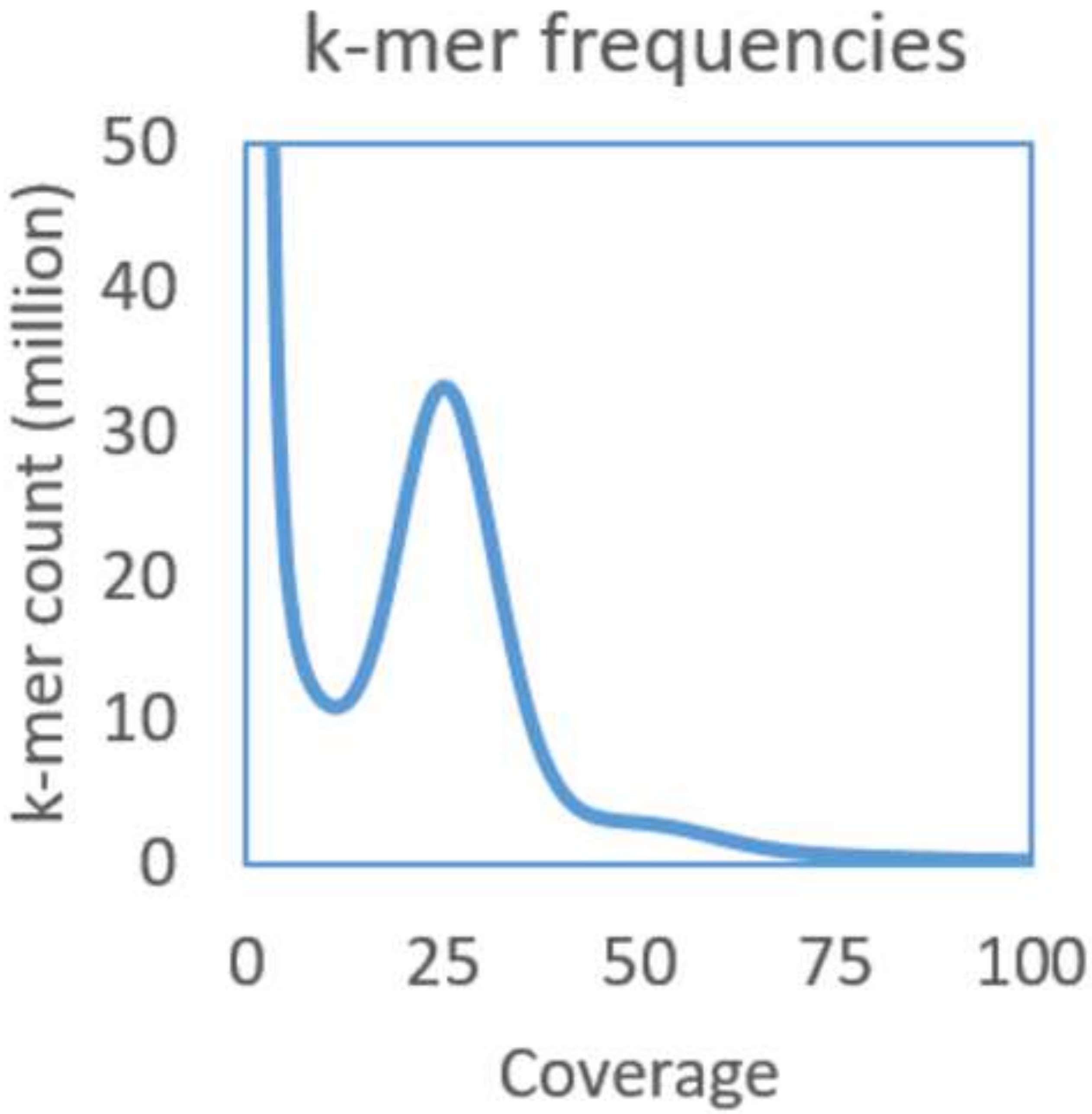
k-mer frequencies
k-mer count (million)
50
40
30
20
10
0
0
25
50
75
100
Coverage

Figure2 Click here to download Figure Figure 2.png

A)

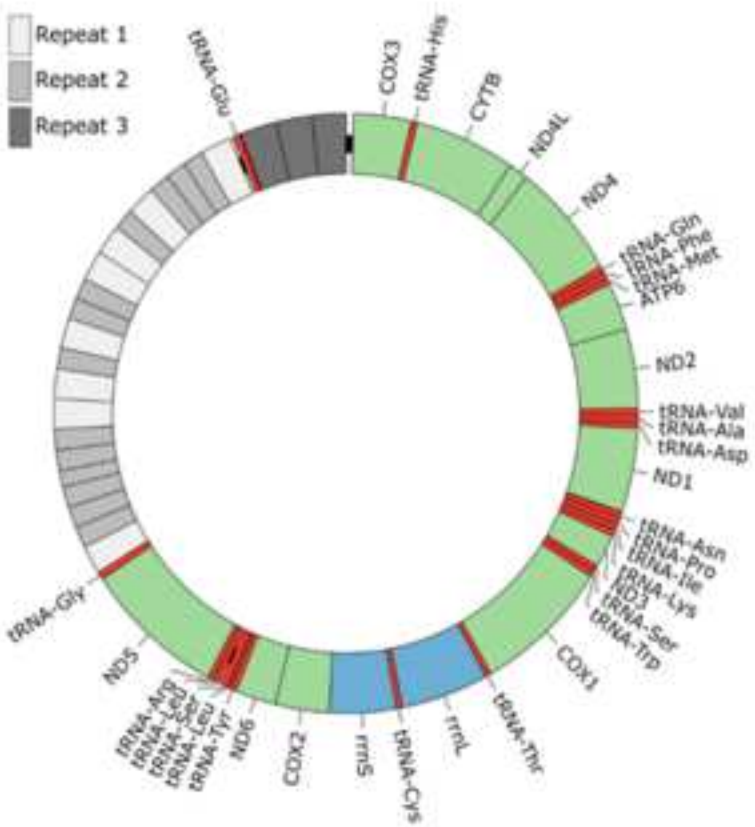

B)

Repeat 1 (328bp ~8 copies)

TGTCAAGTTTGAAGGGACCGATTTAGCTTCGATTCCAATGGGTGTAGAGGTTTGGAGTTGGCGTTGCCTGTTGATTTTCTGTGTCAA
GGGGGGTCTGAAACTATGCGCGAAAAGGGTGCAAAAAAAATTCGTAAGGGGGGGGCATTGCAAACTTTTTCCTTTTTAAAAATTTAC
AGCTTAATTCAGGTCTAGTCGAAGAGTGAAGTGGTTTTTATCTCCCCTTAATTTGACTGTCGATTAAAAATTTTCGTTACTTTTGTG
TCAAAATTACATCATAGCTTTTTTCAGGGGAGTTCGGAGGTGAAAAGTTGGATTTTTGAAGGGTTTG

Repeat 2 (229bp ~13 copies)

TGTCAAGTTTGAAGGGACCGATTTGGCTTCGATTCCAATGGGTGTAGAGGTTTGGAGTTGGCGTTGCCTGTTGATTTTCTGTGTCAA
GGGGGGTTTTAAACTATGCTGTCGAGGGTGTTCACCGTAGCTTTTTTCAGGGGAGTTCGGAGGTGAAAAGTTCGGTTTTTTCGATGA
GCTGGTACGAAATGCTATTATGTTAATCATAAGTAGAGTTATAATTAGAGGTCTC

Repeat 3 (406bp 3 copies)

AAAAAAAGATATCATCGCTAAAAGAGAATAATTGGAAATGACTGTGTTGTCGTAAAGGATTGGGATTAACTGTAGTCCGAGCAGTGT
TGGTTGTGCGAGGAAGAGATGAGGGGGGCTTAATAATAATATGAGGAGGCCCCTACAATGAAAGTGGTTAAGAAGGTCCATTGTAAA
GGATTAGGATTAACTGTAGTCCGAGCAGTGTTGGTTGTGCGAGGAAGAGATGAGGGGGGCTTAATAATAATATGAGGAGGCCCCTAC
AATGAAAGTGGTTAAGAAGGTCCATTGTAAAGGATTAGGATTAACTGTAGTCCGAGCAGTGTTGGTTGTGCGAGGAAGAGATGAGGG
GATCTTTGGTTAATAATACAGGAAGTCATCTGTAATGGGGGGAAAGGGGGGCCTGACC

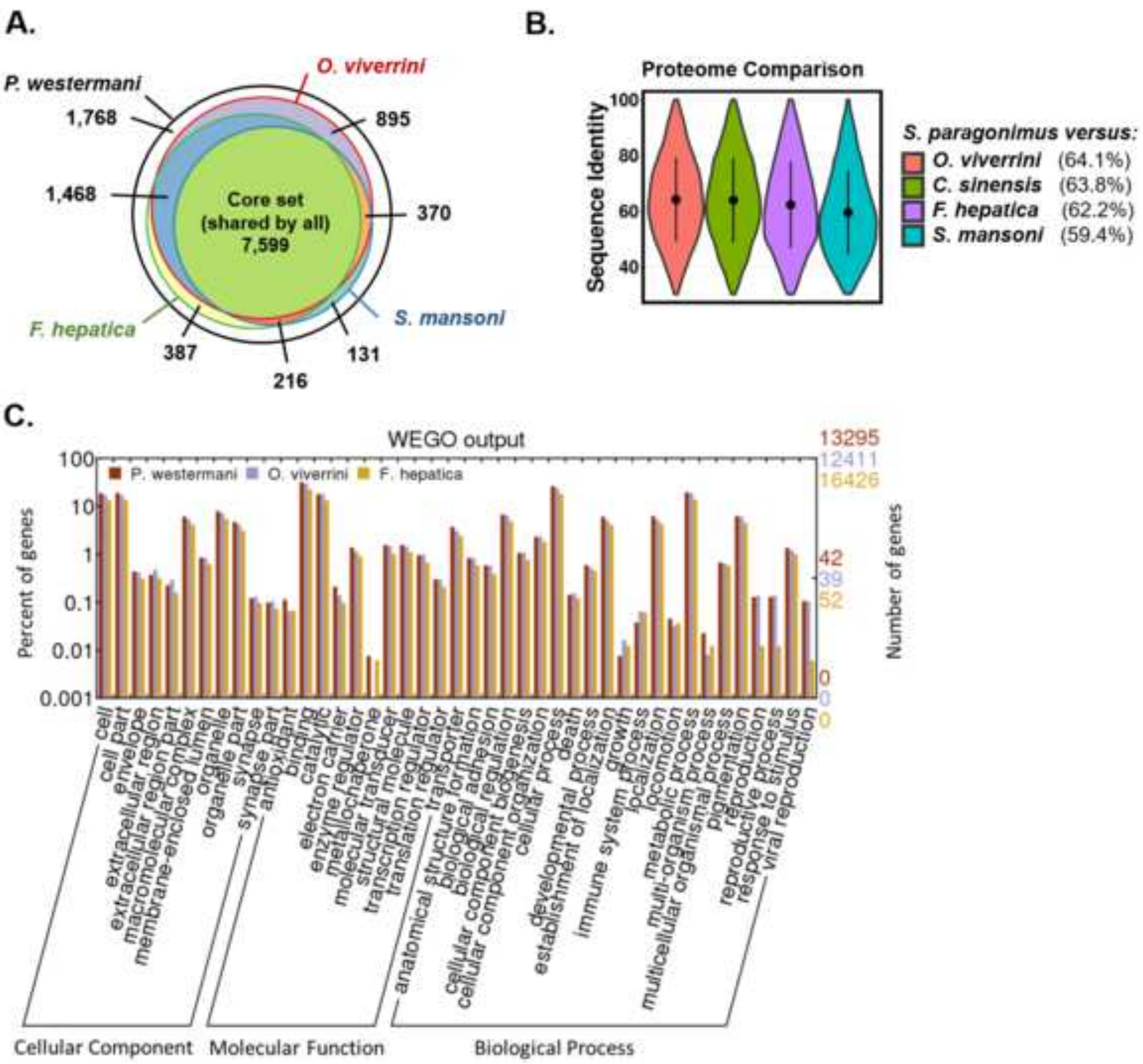
A.
P. westermani
O. viverrini
1,768
895
1,468
Core set
(shared by all)
7,599
370
F. hepatica
S. mansoni
387
131
216
B.
Proteome Comparison
Sequence Identity
100
80
60
40
S. paragonimus versus:
O. viverrini (64.1%)
C. sinensis (63.8%)
F. hepatica (62.2%)
S. mansoni (59.4%)
C.
WEGO output
P. westermani
O. viverrini
F. hepatica
Percent of genes
100
10
1
0.1
0.01
0.001
13295
12411
16426
42
39
52
0
0
0
Number of genes
cell
cell part
envelope
extracellular region
extracellular region part
macromolecular complex
membrane-enclosed lumen
organelle
organelle part
synapse
synapse part
antioxidant
binding
catalytic
electron carrier
enzyme regulator
metallochaperone
molecular transducer
structural molecule
transcription regulator
translation regulator
transporter
anatomical structure formation
biological adhesion
biological regulation
cellular component biogenesis
cellular component organization
cellular process
death
developmental process
establishment of localization
growth
immune system process
localization
locomotion
metabolic process
multi-organism process
multicellular organismal process
pigmentation
reproduction
reproductive process
response to stimulus
viral reproduction
Cellular Component
Molecular Function
Biological Process

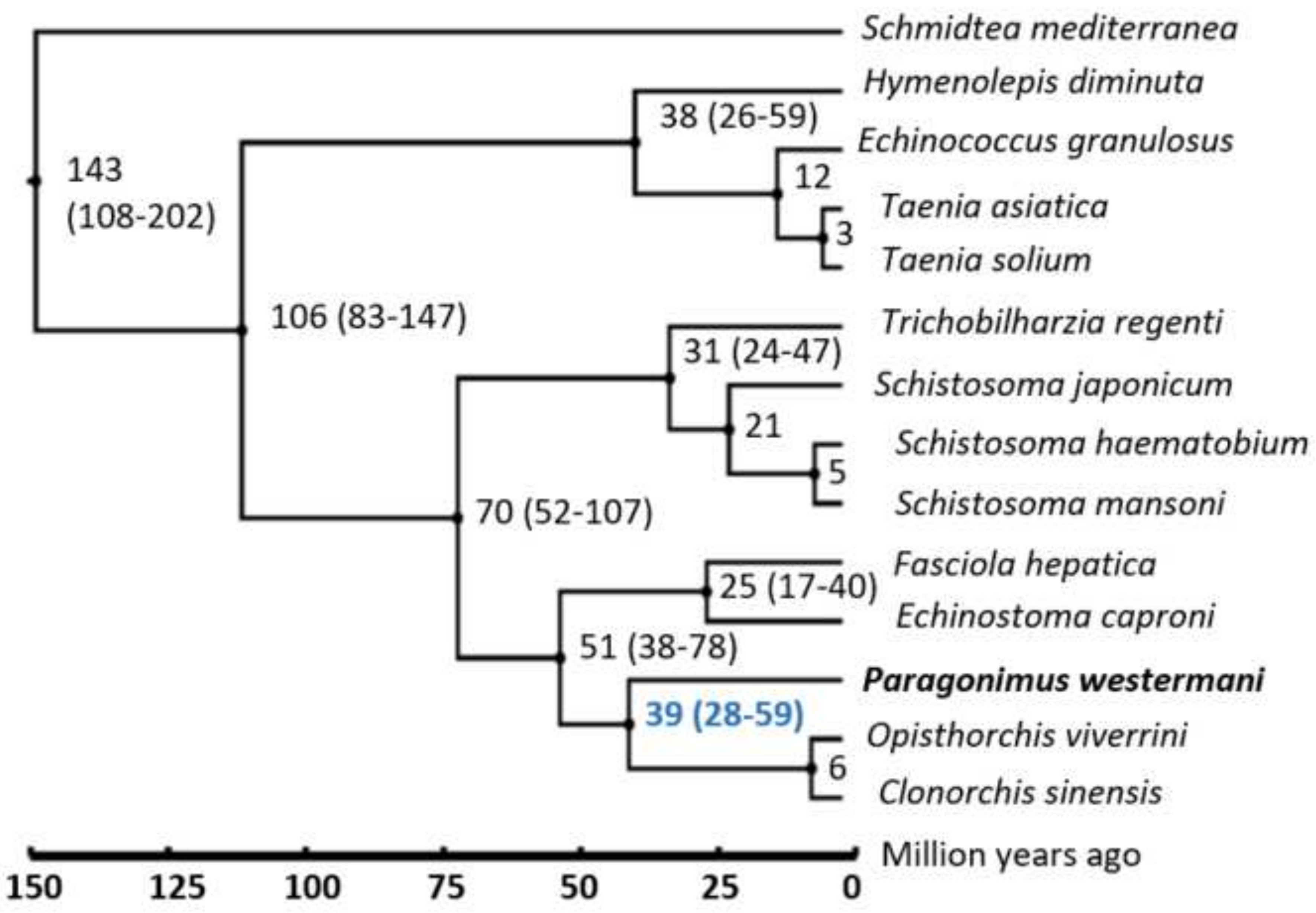
Schmidtea mediterranea
Hymenolepis diminuta
38 (26-59)
Echinococcus granulosus
12
Taenia asiatica
3
Taenia solium
143
(108-202)
106 (83-147)
Trichobilharzia regenti
31 (24-47)
Schistosoma japonicum
21
Schistosoma haematobium
5
Schistosoma mansoni
70 (52-107)
Fasciola hepatica
25 (17-40)
Echinostoma caproni
51 (38-78)
Paragonimus westermani
39 (28-59)
Opisthorchis viverrini
6
Clonorchis sinensis
Million years ago
150
125
100
75
50
25
0
