# Supplementary material for: Whole-genome sequence of the oriental lung fluke Paragonimus westermani
Source: Gigascience. 2018 Dec 6;8(1):giy146. doi: 10.1093/gigascience/giy146 (PMC6329441; doi:10.1093/gigascience/giy146)
Supplement: GIGA-D-18-00193_Revision_1.pdf [file giy146_giga-d-18-00193_revision_1.pdf]

GigaScience

Whole-genome sequence of the oriental lung fluke Paragonimus westermani

--Manuscript Draft--

**Manuscript Number:**	GIGA-D-18-00193R1
**Full Title:**	Whole-genome sequence of the oriental lung fluke Paragonimus westermani
**Article Type:**	Data Note
**Funding Information:**	the QIMR Berghofer Medical Research Institute (Chenhall Estate) — A/Prof Lutz Krause<br>Australian Infectious Diseases Research Centre — A/Prof Lutz Krause<br>National Health and Medical Research Council — Prof Donald P. McManus

Abstract:

Background

Foodborne infections caused by lung flukes of the genus Paragonimus are a significant and widespread public health problem in tropical areas. Around 50 Paragonimus species have been reported to infect animals and humans, but Paragonimus westermani is responsible for the bulk of human disease. Despite their medical and economic importance, no genome sequence for any Paragonimus species is available.

Results

We sequenced and assembled the genome of P. westermani, which is among the largest of the known pathogen genomes with an estimated size of 1.1 Gb. A 922.8 Mb genome assembly was generated from Illumina and PacBio sequence data, covering 84% of the estimated genome size. The genome has a high proportion (45%) of repeat-derived DNA, particularly of the LINE and LTR subtypes, and the expansion of these elements may explain some of the large size. We predicted 12,852 protein coding genes, showing a high level of conservation with related trematode species. The majority of proteins (80%) had homologs in the human liver fluke Opisthorchis viverrini with an average sequence identity of 64.1%. Assembly of the P. westermani mitochondrial genome from long PacBio reads resulted in a single high-quality circularized 20.6 kb contig. The contig harboured a 6.9 kb region of non-coding repetitive DNA comprised of three distinct repeat units. Our results suggest that the region is highly polymorphic in P. westermani, possibly even within single worm isolates.

Conclusions

The generated assembly represents the first Paragonimus genome sequence and will facilitate future molecular studies of this important, but neglected, parasite group.

**Corresponding Author:**	Lutz Krause<br><br>AUSTRALIA
**Corresponding Author Secondary Information:**	
**Corresponding Author's Institution:**	
**Corresponding Author's Secondary Institution:**	
**First Author:**	Harald Oey
**First Author Secondary Information:**	
**Order of Authors:**	Harald Oey
	Martha Zakrzewski
	Kanwar Narain
	K. Rekha Devi
	Takeshi Agatsuma
	Sujeevi Nawaratna

Powered by Editorial Manager® and ProduXion Manager® from Aries Systems Corporation

	Geoffrey Gobart
	Malcolm Jones
	Mark A. Ragan
	Donald P. McManus
	Lutz Krause
**Order of Authors Secondary Information:**	
**Response to Reviewers:**	Reviewer: 1<br>1 – I suggest a small change in the manuscript title: "Draft Whole genome sequence of the oriental lung fluke…" or just "Whole genome sequence of the …". The term complete for nuclear genome sequence means that it is the final version (in chromosome level with no gaps), not the case here where the genome is still in 30,977 pieces, so complete should be not used here. The mitochondrial indeed looks complete.<br>Response: We have changed the manuscript title to “Whole-genome sequence of the oriental lung fluke Paragonimus westermani” as suggested by the reviewer.<br><br>2 – The authors did not mention how they removed potential contamination or how they maintained the pathogen for the DNA extraction (Please add this information)<br>Response: Comparison of assembled scaffolds with public genome sequence data identified contamination by rat (the experimental host) and the bacterium Delftia sp. All sequences mapped to these genomes were removed. We have now added this information to the methods section.<br><br>3 – Table 1 could be used as supplemental material<br>Response: We intend to submit the manuscript as Data Note. We believe that Table 1 is important for a Data Note and suggest we keep it in the main manuscript.<br><br><br>4 – The assembly was performed by well-known genome assemblers, but there was any particular reason to not use any of the two most used PacBio assemblers (HGAP and CANU?)<br>Response: We have used CANU for several parasite genomes. The program worked well for other parasites (manuscript in review), but did not perform well on this particular genome. Mira worked better for Paragonimus and generated a single complete mitochondrial contig, whereas CANU resulted in multiple shorter contigs.<br><br><br>5 – The authors choose to use for the Illumina assembly the ABYSS assembler. From my personal experience and from some colleagues there are several other assemblers that give a better job than ABYSS (Spades, MIRA, Velvet and SoapDenovo2). I know that it varies depending of the nature of the organism and sample used for the assay, but since the group used for the gapfilling step the soapDenovo gapcloser, I would like to see in the manuscript some information about why these pipelines were choosen beside others<br>Response: We have evaluated several assembly programs and ABYSS performed best for this particular genome. ABYSS is also one of the few assemblers that allow inclusion of long-read data to guide scaffolding. The program is still widely used and well maintained. We have an established pipeline using SoapDenovo2, which has been used for the assembly of other parasite genomes (manuscript in review). However, SoapDenovo2 did not perform well for this particular genome, with a large size and many repetitive regions. Additionally, the Paragonimus genome was sequenced from 50 individual worms, resulting in a low-level sequence heterogeneity and assembly of this data proofed to be challenging. ABYSS performed particularly well for the assembly of contigs for this genome. However, the ABYSS gap filler is not well suited for closing gaps larger than 1kb (according to the ABYSS manual and our own experience), whereas the soapDeNovo gapfiller is well suited for this task and performed particularly well on this genome. Additional information has been added to the methods section.<br><br>6 – Line 179 - REAPR typo. I would also suggest the authors to perform for this final polishing genome correction step Pilon or ICORN2 using the Illumina reads generated

Response: We thank the reviewer for this suggestion. However, Pilon does not seem to perform well for this particular genome. Genome polishing using Pilon with a variety of different settings actually resulted in a slight reduction of BUSCO scores (original assembly: 65.3% complete proteins; after Pilon: 63.9% complete proteins), indicating that Pilon did not improve the overall quality of this particular genome assembly. We manually investigated Pilon results and postulate that Pilon was misled by low-level sequence heterogeneity caused by the pooling of 50 individual worms. As the genome has already been deposited in NCBI and passed all manual QC checks we believe that the questionable improvements by Pilon do not justify re-submission of an updated genome to NCBI.

7 – Please add more information about the genome assembly statistics in table 2 (L50 and number of Ns), a quick run on QUAST should give you this information. And please explain if these gaps are just generated during the scaffolding by the mate pair evidence or it was also generated for unknown size gaps (100Ns). This information is really important to show that some regions could be missing in this draft genome assembly, so future studies could be aware of this fact;
Response: We have run the assembly through QUAST, as requested, and added the L50 to Table 2. The number of Ns can already be inferred from Table 2 as we provide the size of the genome both with and without counting Ns ("Assembly size" and "Total base pairs"). We have re-named "Total base pairs" to "Ungapped size" to make this clearer. The Gaps are generated both during contig assembly (abyss) and scaffolding (SSPACE) and represent the estimated size of the gaps. We have added a sentence to the manuscript to make this clear.

8 – Line 250 - Since the ncRNA information was so important in the mitochondrial annotation, and the group already characterized the tRNAs, please add the method to predict these tRNAs (like tRNAscan) and also, I suggest adding an Aragorn or inferno ncRNA prediction run to improve even more the annotation
Response: The program Mitos, which was used to characterize the mitochondrial genome, identifies both non-coding RNAs and proteins. However, Aragorn was also run to identify any additional tRNAs in the mitochondrial genome (added to methods).

9 – Line 258 - no problem with the methodology, but Cufflinks has a substitute, StringTie (Petera at el., 2015). It will do a much better job to assembly the transcriptome
Response: StringTie was not available when the project started, but we thank the reviewer for this suggestion and will evaluate StringTie for future projects. Cufflinks is well established (>5,000 citations), proven to generate accurate results and is still widely used. We agree that there are many alternative tools that could have been used for transcriptome assembly, but our group has an established and well tested pipeline using cufflinks. We have extensive experience with cufflinks and have optimized the parameters to generate robust and high-quality results. We would further like to point out that we don't publish the assembled cDNA data.

10 – Genome Comparison - I understand that this was not the focus of this manuscript, but sequence identity besides important is a too general comparison method. I suggest add a orthology analysis and maybe generate a Circos synteny plot comparing the new genome with the most similar species available
Response: We will submit the manuscript as Data Note and therefore believe that additional comparative analysis are not required.

11 – Phylogeny - Add a Modeltest run to check if Jones-Taylor-Thornton (JTT) was the best substitution method to be used. For the ML analysis I suggest using PhyML instead of Phylip again, the software used is good but better and newer ones were developed
Response: As suggested, we have now repeated the phylogenetic analysis using PhyML and a model test found the LG substitution model with decorations +G+I+F as optimal. The JTT model was the second best model. PhyML using the LG+G+I+F

model resulted in exactly the same tree topology as our previous analysis using Phylip with the JTT model, demonstrating the robustness of our inferred phylogenetic relationships.

12 – Bayesian method - MCMCTREE in PAML is good, but since Bayesian methods tend to vary, I suggest the group to run another test using the most known softwares (BEAST or mrBayes), to check if these mrca inferences are matching properly
Response: As suggested, we have now estimated divergence times using BEAST version 2. BEAST v2 estimates matched our previous results from MCMCTREE well and were within the estimated confidence intervals. Divergence times estimated by BEAST v2 were added to Figure 4 of the manuscript.

13 – Figure 1 - Doesn't need to be a main figure. Could be used as supplementary figure.
Response: The manuscript has been changed to Data Note and we believe that Figure 1 is important for this manuscript type.

14 – Figure 2 B - These sequences could be mentioned in the text and added as supplementary file. You can name these repeats if needed in figure 2 A.
Response: We agree and have moved the text to the supplementary data.

15 – Figure 3 - Figure is fine but needs to improve image quality. It is preferable to have a Venn diagram of the orthologs between these species.
Response: We have now replaced the figure with a non-proportional Venn diagram.

16 – Add a circus synteny plot figure between the new genome and the closest species genome available.
Response: We have re-submitted the manuscript as Data Note and we believe that in this case a synteny plot is not needed. Additionally, while we agree that a synteny plot would be valuable, generating a synteny plot would be problematic for the Paragonimus genome, as no close relative genome of high quality is available that would allow ordering of the scaffolds.

17 – Figure 4 - (optional) Try to make the same figure using Figtree. They have a nicer way to show the median of the mrca on each node.
Response: We have now improved the figure and aligned the numbers with the tree branches.

Reviewer 1 minor comments:
18 – Change the word faeces for stool. It's not wrong, but stool is more commonly used worldwide;
Response: We have changed “faeces” to “stool” as suggested.

19 – Line 148 - Data Sequencing: add the Illumina Platform used in the data generation (example: HiSeq2000)
Response: Done as suggested.

20 – Line 150 - Data Sequencing: add the PacBio Platform used in the data generation (example: PacBio Sequel or RSII)
Response: Done as suggested.

Reviewer: 2
The manuscript is currently written in my opinion as a data note rather than a research type manuscript. If this is the intention this should be made clearer by the authors as part of their submission. If the manuscript is intended to be submitted as a research paper, the authors should expand on their discussion and conclusions of their data.

Powered by Editorial Manager® and ProduXion Manager® from Aries Systems Corporation

Response: We have resubmitted the manuscript as a Data Note.

1 – Abstract, line 85 and Data description, line 157: The authors computationally determined the estimated size of the P.westermani genome, prior to assembly of the raw reads. The computationally determined estimated size was slightly larger than the assembled genome size. The authors should comment on the size difference. In addition, the authors interchange throughout the manuscript whether they compare the estimated size or the assembled genome size with other known published trematode genomes. Until it can be shown that the genome of P.westermani is actually 1.1 Gb, the authors should only refer to the assembled genome size particularly in the section around line 157, as these published trematode genomes describe only the assembled genome sizes.
Response: As suggested, we have added a comment regarding the genome size differences and now base the genome size comparison on the assembled genome sizes.

2 – Line 144 - at what point of infection were the parasites recovered - specifically how old were the parasites?
Response: The parasites were 30-40 days old, this information has been added to the manuscript.

3 – Lines 144-146 -Further information is required regarding the methodology of genomic DNA extraction. Was the extraction carried out on individual worms and then combined or were the worms combined for extraction? Was the genomic DNA quality checked?
Response: The following information has been added to the manuscript: “Genomic DNA was isolated from a pool of 50 worms (30 – 40 days of age), yielding 18 µg of DNA. DNA was quantified by Pico green, QUBIT and NanoDrop. Degradation was tested by Microplate Reader and Agarose Gel Electrophorese (concentration of agarose gel: 1%, electrophoresis time: 40 min, voltage 150 V).

4 – Line 150 - can the authors confirm that the PacBio sequencing was performed on the same sample of genomic DNA?
Response: We confirm that the same sample of genomic DNA was used for PacBio and Illumina sequencing.

5 – Line 255 - the authors should mention that the RNAseq data was from adult parasites only, not the various different lifecycle stages.
Response: This information has now been added to the manuscript.

6 – Line 221 - Related to point 3, as the authors extracted DNA from 50 individual worms, did they check the level of polymorphism at the individual worm level for this region?
Response: We agree with reviewer that this would be an interesting question. However, DNA was isolated from a pool of 50 individual worms. Moreover, only 5 reads spanned the region in full (anchored in non-repetitive sequence at both ends), which was sufficient to generate a consensus sequence for the region, but not to accurately quantify individual-level differences.

7 – Line 272-273 - If the authors are submitting a research themed manuscript, they could include some further discussion of the predicted protein coding genes, particularly those predicted proteins that have inferred homologs in other trematodes (Fig 3A) and the Paragonimus-specific predicted proteins.
Response: The manuscript has been re-submitted as Data Note.

8 – Figure 3A - the venn diagram is currently difficult to interpret, particularly given its current small size as a multi-panel figure. I suggest amending this figure to a classical

	venn diagram or an Upset plot.<br>Response: The figure has been replaced by a non-proportional Venn diagram.<br><br><br>9 – The authors should include supplemental data detailing the functional annotation particularly the analysis of the functional domains, transmembrane domains and signal peptides, as well the data relating to the single copy predicted proteins used for the phylogenetic analysis.<br>Response: As requested, we have now uploaded our InterProScan results as well as the sequences for single copy proteins used for the phylogenetic analysis to the GigaScience ftp server.<br><br><br>10 – Minor corrections:<br>a.line 118 - develop into sporocysts<br>b.line 161 - 1.3 Gb<br>c.line 204, 291, 293 - BLAST<br>d.line 281 - predicted proteome<br>Response: We thank the reviewer for these comments. The manuscript has been modified as suggested.
**Additional Information:**	
**Question**	**Response**
Are you submitting this manuscript to a special series or article collection?	No
**Experimental design and statistics**<br><br><br>Full details of the experimental design and statistical methods used should be given in the Methods section, as detailed in our Minimum Standards Reporting Checklist. Information essential to interpreting the data presented should be made available in the figure legends.<br><br><br>Have you included all the information requested in your manuscript?	Yes
**Resources**<br><br><br>A description of all resources used, including antibodies, cell lines, animals and software tools, with enough information to allow them to be uniquely identified, should be included in the Methods section. Authors are strongly encouraged to cite Research Resource Identifiers (RRIDs) for antibodies, model organisms and tools, where possible.<br><br><br>Have you included the information	Yes

requested as detailed in our Minimum Standards Reporting Checklist?	
**Availability of data and materials**<br><br>All datasets and code on which the conclusions of the paper rely must be either included in your submission or deposited in publicly available repositories (where available and ethically appropriate), referencing such data using a unique identifier in the references and in the “Availability of Data and Materials” section of your manuscript.<br><br>Have you have met the above requirement as detailed in our Minimum Standards Reporting Checklist?	Yes

Manuscript

Click here to view linked References

Click here to access/download;Manuscript;Paragonimus_GigaSciences_Re

Title Page

Whole-genome sequence of the oriental lung fluke *Paragonimus westermani*

Harald Oey[1, #], Martha Zakrzewski[2], Kanwar Narain[6], K. Rekha Devi[6], Takeshi Agatsuma[7], Sujeevi Nawaratna[2], Geoffrey Gobart[2,4], Malcolm Jones[3], Mark A. Ragan[5], Donald P. McManus[2,*], Lutz Krause[1,2,*,#]

[1] The University of Queensland Diamantina Institute, Brisbane, QLD, Australia

[2] QIMR Berghofer Medical Research Institute, Brisbane, QLD, Australia

[3] School of Veterinary Science, University of Queensland, Gatton, QLD, Australia

[4] School of Biological Sciences, Queen's University Belfast, UK

[5] Institute for Molecular Bioscience, The University of Queensland, Brisbane, QLD, Australia

[6] Regional Medical Research Centre for Northeastern region, Indian Council of Medical Research Department of Health Research Ministry of H & FW, Govt. of India, Dibrugarh-786 001, India

[7] Department of Environmental Medicine, Kochi Medical School, Kochi University, Kohasu, Oko, Nankoku City Kochi 783-8505

[*] authors contributed equally

[#] To whom correspondence should be addressed

Contact: l.krause@uq.edu.au and h.oey@uq.edu.au

Author names, affiliations and emails:

Dr Harald Oey

The University of Queensland Diamantina Institute, The Faculty of Medicine, The University of Queensland, Translational Research Institute, Brisbane, QLD

Email: h.oey@uq.edu.au

Dr Martha Zakrzewski

QIMR Berghofer Medical Research Institute, Brisbane, QLD, Australia

Email: Martha.Zakrzewski@qimrberghofer.edu.au. ORCID: 0000-0003-1436-5070.

Dr Kanwar Narain

Regional Medical Research Centre for Northeastern region, Indian Council of Medical Research Department of Health Research Ministry of H & FW, Govt. of India, Dibrugarh-786 001, India

Email: kanwar_narain@hotmail.com

Dr K. Rekha Devi

Regional Medical Research Centre for Northeastern region, Indian Council of Medical Research Department of Health Research Ministry of H & FW, Govt. of India, Dibrugarh-786 001, India

Email: krekha75@yahoo.co.in

Prof Takeshi Agatsuma

Department of Environmental Medicine

Kochi Medical School, Kochi University, Kohasu, Oko, Nankoku City Kochi 783-8505

Email: agatsuma@kochi-u.ac.jp

Dr Geoffrey Gobert

School of Biological Sciences, Queen's University Belfast, UK

Email: g.gobert@qub.ac.uk

Prof Mark A. Ragan

Institute for Molecular Bioscience, The University of Queensland, Brisbane, QLD, Australia

Email: m.ragan@uq.edu.au. ORCID: 0000-0003-1672-7020

Prof Malcolm Jones

School of Veterinary Science, University of Queensland, Gatton, QLD, Australia

Email: m.jones@uq.edu.au

Dr Sujeevi Nawaratna

QIMR Berghofer Medical Research Institute, Brisbane, QLD, Australia

Email: sujeevi.nawaratna@qimrberghofer.edu.au. ORCID: 0000-0001-8716-2296

Prof Donald P. McManus

QIMR Berghofer Medical Research Institute, Brisbane, QLD, Australia

Email: Don.McManus@qimrberghofer.edu.au

A/Prof Lutz Krause

The University of Queensland Diamantina Institute, The Faculty of Medicine, The University of Queensland, Translational Research Institute, Brisbane, QLD

Email: L.Krause@uq.edu.au. ORCID: 0000-0003-3806-0845

Abstract

Background

Foodborne infections caused by lung flukes of the genus *Paragonimus* are a significant and widespread public health problem in tropical areas. Around 50 *Paragonimus* species have been reported to infect animals and humans, but *Paragonimus westermani* is responsible for the bulk of human disease. Despite their medical and economic importance, no genome sequence for any *Paragonimus* species is available.

Results

We sequenced and assembled the genome of *P. westermani*, which is among the largest of the known pathogen genomes with an estimated size of 1.1 Gb. A 922.8 Mb genome assembly was generated from Illumina and PacBio sequence data, covering 84% of the estimated genome size. The genome has a high proportion (45%) of repeat-derived DNA, particularly of the LINE and LTR subtypes, and the expansion of these elements may explain some of the large size. We predicted 12,852 protein coding genes, showing a high level of conservation with related trematode species. The majority of proteins (80%) had homologs in the human liver fluke *Opisthorchis viverrini* with an average sequence identity of 64.1%. Assembly of the *P. westermani* mitochondrial genome from long PacBio reads resulted in a single high-quality circularized 20.6 kb contig. The contig harboured a 6.9 kb region of non-coding repetitive DNA comprised of three distinct repeat units. Our results suggest that the region is highly polymorphic in *P. westermani*, possibly even within single worm isolates.

Conclusions

The generated assembly represents the first *Paragonimus* genome sequence and will facilitate future molecular studies of this important, but neglected, parasite group.

Keywords

Paragonimus westermani, whole-genome sequence, genome assembly, paragonimiasis, food-borne disease, oriental lung fluke, parasitic infection, bioinformatics, high-throughput sequencing, comparative genomics, genome annotation, neglected tropical disease, flatworm

Background

Paragonimus lung flukes represent a significant and widespread clinical problem with an estimated 23 million people infected worldwide (1). Around 50 species are described, with at least 7 being human pathogens (2). The majority of human *Paragonimus* infections can be attributed to the *P. westermani* species complex, mainly in Southeast Asia and Japan (1). *P. westermani* show considerable geographic genetic variability and human infections occur predominantly in East Asia and the Philippines. In India the incidence rates of paragonimiasis caused by *P. westermani* is currently unknown(2-4), however many cases of paragonimiasis are attributed to the related worm *Paragonimus heterotremus* (2). Paragonimiasis is a zoonotic disease and also pigs, dogs and other animals can harbour *P. westermani* (2).

Paragonimus spp. have a complex life cycle. Unembryonated eggs are expelled by coughing or passed with stool and develop in water. Miracidia hatch from the eggs and penetrate a freshwater snail, its first intermediate host. During several asexual developmental phases inside the snail, a miracidium develops into a sporocyst and then two redial generations occur, the second of which gives rise to microcercous cercariae that escape into fresh water. These crawling cercariae invade a species of crustacean, the second intermediate host, to encyst in muscles and other sites and develop into metacercariae. Humans and other definitive hosts become infected through consumption of raw or inadequately cooked freshwater crabs or crayfish (5). Ingested metacercariae excyst, penetrate through the gut and become encapsulated in the lungs where they mature into hermaphroditic adult worms (7.5 mm to 12 mm in length) in 6-10 weeks (5). Paragonimiasis can lead to a chronic inflammatory disease of the lung and can trigger asthma- or tuberculosis-like symptoms (6-8). In more severe cases *Paragonimus* can infect the brain or central nervous system of the definitive host, leading to headache, visual loss, and even death (1).

Paragonimiasis is commonly diagnosed by microscopic detection of parasite eggs in stool or sputum. The lack of sensitive and reliable diagnostic tests in conjunction with unspecific disease symptoms often leads to delayed treatment with the drug of choice, praziquantel (8). Despite their high medical, veterinary and economic importance, only limited information on the molecular biology of *Paragonimus* is currently available. Recent

transcriptome sequencing studies have provided some information on the gene content of *Paragonimus* (9), but until now no *Paragonimus* genome sequence has been available. Here we present a 922.8 Mb assembly of the *P. westermani* genome which provides new insights into the genomic composition of the *Paragonimus* genus and represents an invaluable resource for future studies of the neglected tropical disease paragonimiasis.

Data Description

Sequencing

Diploid *Paragonimus westermani* metacercariae (NCBI:txid34504) were collected from the freshwater crab *Maydelliathelphusa lugubris* in 2009 in the East Siang district of Arunachal Pradesh, Northeast India, and fed to Wistar rats as experimental hosts. Genomic DNA was isolated from a pool of 50 worms (30 – 40 days of age), yielding 18 µg of DNA. DNA was quantified by Pico green, Qubit and NanoDrop and degradation was tested by Microplate Reader and Agarose Gel Electrophorese (concentration of agarose gel: 1%, electrophoresis time: 40 min, voltage 150 V). The *Paragonimus westermani* genome was then sequenced from 2 µg of the isolated DNA using a whole-genome shotgun approach. Paired-end short-insert (200 bp and 450 bp) and mate-pair (5 Kb and 10 Kb) genomic DNA libraries were sequenced on the Illumina HiSeq 2000 platform, yielding 58 Gb of sequence data (**Table 1**). For genome scaffolding and quality evaluation of the assembled sequence, additional long-read data were generated from the same genomic DNA sample using the PacBio RSII platform, yielding 1.7 Gb of information (**Table 1**). The genome size was estimated from the K-mer coverage of the 450 bp insert library. K-mer frequencies were calculated by the program Jellyfish (10), version 2.2.6 , using a K-mer size of 17bp. The 17-mer distribution in the 450 bp library had a single peak at 26x (**Figure 1**), demonstrating low sequence heterozygosity. The genome size (G) was deduced from the K-mer distribution via the formula $G = N * (L – K + 1) / K\_depth$(11), where N is the total number of reads, L is the read length, K is the K-mer size and K_depth is the peak frequency. The *P. westermani* genome size was estimated to be 1.1 Gb.

Table 1. *Paragonimus westermani* sequencing libraries.

Library	Platform	Library type	Insert size (bp)	Read length (bp)	Read count (raw)
200bp	HiSeq	Paired-end	200	2 x 120	140,542,299
450bp	HiSeq	Paired-end	450	2 x 100	171,954,230
5kb	HiSeq	Mate-pair	5,000	2 x 49	232,630,904
10kb	HiSeq	Mate-pair	10,000	2 x 49	266,480,540
PacBio	PacBio	Long read	-	-	1,731,327

Genome assembly

PacBio sequence data were error corrected by proovread version 2.13.13 (12), using Illumina short reads from the 200bp and 450bp libraries as input , and assembled into contigs by Mira v4.0.2 (MIRA, RRID:SCR_010731)(13). Short-read Illumina sequence data were trimmed using Trimmomatic v0.36 (Trimmomatic , RRID:SCR_011848) and subsequently error corrected by KmerFreq_HA (part of SoapDenovo2 (14)) with a K-mer size of 23. The 10 kb mate-pair library showed a high proportion of PCR duplicates and was subjected to PCR de-duplication prior to genome assembly. For assembly of short read data, several assembly programs were evaluated. ABYSS performed best for this particular genome with its large size, high percentage of repetitive regions and some low-level sequence heterogeneity resulting from pooling genomic DNA from the 50 individual worms. ABYSS is also one of the few assemblers that allow inclusion of long-read data to guide scaffolding. Illumina paired-end sequence data were assembled using the ABYSS assembly pipeline (ABySS , RRID:SCR_010709)(15), version 2.0.2, with options n=5 s=200 N=36 S=500 k=33 and including the PacBio contigs via the re-scaffolding feature.

The resulting assembly was de-gapped using the SoapDenovo2 GapCloser program (GapCloser, RRID:SCR_015026)(14) which is well suited for closing gaps larger than 1kb and it performed particularly well on this genome. Mate-pair libraries were then used to scaffold the assembly with SSPACE v3.0(16) (with options -x 0 -a 0.60 -n 30 -z 200 -g 0) and gaps were again filled with GapCloser. Un-closed gaps are represented by N's spanning the estimated sizes of the gaps. To detect and resolve scaffolding errors, the resulting assembly was processed by the program REAPR (17) using the 5kb mate-pair library as input, breaking

the assembly at sites with poor evidence for contiguity. Contamination due to the experimental rat host and the bacterium *Delftia* sp was detected based on a comparison of predicted proteins with the NCBI protein database using BLAST and, additionally, via the NCBI Genome Submission Portal quality control pipeline. A targeted comparison of all scaffolds with the genomes of the rat and *Delftia* using BLAT identified 531 short scaffolds with high similarity (>90%) to these genomes. These sequences were manually scrutinized, with 529 of the affected scaffolds found to be completely derived from rat or *Delftia*, and these were removed from the assembly. The remaining two contaminated sequences represented rat ribosomal DNA that had been erroneously incorporated into *Paragonimus* scaffolds and were also removed from the final assembly by cutting and trimming the affected scaffolds.

The final assembly resulted in a 922.8 Mb genome sequence (30,466 scaffolds with N50 of 135 Kb) (**Table 2**), covering 84.0% of the estimated genome size. The discrepancies in genome size can potentially be the result of problematic DNA regions that are difficult to sequence or assemble (e.g. regions with strong secondary structures, highly repetitive regions or long homopolymeric runs) or the result of low-level sequence heterogeneity which can lead to an overestimation of genome size by k-mer approaches. The *P. westermani* genome sequence is among the largest known pathogen genomes and one of the largest parasite genomes sequenced to date. The assembled genome sequence is considerably larger than the published genomes of the related trematodes *Clonorchis sinensis* (assembly size of 546.9 Mb) (18), *Opisthorchis viverrini* (606.0 Mb) (19), and *Schistosoma* spp (364.5-397.7 Mb) (20-22) and comparable to the 1.3 Gb genome of *Fasciola hepatica* (23).

The GC content of the genome was 43.3%, comparable to genomes of other related trematodes (**Table 2**). Genome assembly completeness was evaluated by BUSCO (BUSCO , RRID:SCR_015008)(24) using the metazoan lineage data, resulting in similar scores to those obtained for the genomes of comparable trematode species (**Table 2**). The proportion of duplicated genes reported by BUSCO was also similar to that of comparable trematodes suggesting that the relatively large size of the *P. westermani* genome is not the result of genome duplication events.

Table 2. Assembly statistics for *P. westermani* and comparable trematode genomes of similar size.

	P. westermani	*F. hepatica*	*O. viverrini*	*C. sinensis*
Assembly size (Mb)[a]	922.8	1,275.0	606.0	546.9
Ungapped size (Mb)[b]	877.7	1,183.5	558.0	547.1
Contig N50 (Kb)	7.0 (>100bp)	9.7	NA	14.7
Scaffold N50 (Kb)	135 (>1Kb)	204	1,324	30.2
Scaffold L50	1,943	1,799	135	408
Scaffold count	30,466 (>1Kb)	45,354 (>1kb)	4,919 (>1kb)	31,822
GC content (%)	43.3	44.1	43.8	44.1
Repeat content (%)	45.2	57.1	28.9	32.6
Protein coding genes	12,852	15,740[c]	16,356	13,634
Longest scaffold (Kb)	809	1,565	9,657	2,050
BUSCOS - Complete	65.3%	65.8%	71.4%	70.8%
BUSCOS - Duplicated	1.4%	0.8%	1.1%	1.5%
BUSCOS - Missing	25.8%	25.4%	23.0%	23.1%

[a]Combined length of all scaffolds in Mb. [b]Combined length of all scaffolds without gaps (N's) in Mb. [c]Non-overlapping RNAseq-supported gene models(23)

Mitochondrial genome

The mitochondrial genome of *P. westermani* is present at a much higher copy number than the nuclear genome and we were able to assemble the full mitochondrial genome at high coverage from error corrected long PacBio reads using the Mira assembler(13), version 4.0.2. This resulted in a single mitochondrial contig of 20.3 Kb (**Figure 2**). The accuracy of the contig was confirmed by mapping short insert paired-end sequences directly onto the contig revealing single nucleotide discrepancies at only 4 positions. The mitochondrial genome was found to closely match previously published *Paragonimus* mitochondrial genomes with the best match from a BLAST search against the Nucleotide collection at the NCBI (https://blast.ncbi.nlm.nih.gov/Blast.cgi) being accession NC_027673.1, a *P. westermani*

complex sp. type 1 mitochondrial genome isolated in India (97% sequence identity across 13.4 Kb of NC_027673.1). This sequence was used as reference for mitochondrial gene identification and annotation, supplemented by mitochondrial gene predictions by Mitos (25) and tRNA prediction by Aragorn (Aragorn, RRID:SCR_015974)(26). The mitochondrial genomes of flatworms are known to harbour a region of non-coding repetitive DNA, generally comprised of a long noncoding region (LNR) and a short non-coding region (SNR) with a single tRNA gene separating them(27). Reconstructing this region from short-read data proved challenging, but our long-read PacBio data allowed complete assembly of the repetitive region and circularization of the genome. Interestingly, our assembled mitochondrial genome sequence had a much longer non-coding region (6.9 Kb) than the previously published NC_027673.1 (0.7 Kb) and the non-coding regions of both genomes showed only partial homology, but with close homology of the intervening tRNA gene. We found the LNR to be comprised of two distinct repeat units with 8 and 13 copies while the SNR was comprised of another distinct repeat unit with 3 copies (**Figure 2 and Additional File 1**). Strikingly, five independent PacBio reads spanned the entirety of the non-coding region but with slight differences in length (6.3 – 6.9 Kb), suggesting that the region is polymorphic, possibly even within individual worms.

Repeat annotation

RepBase repeat consensus sequences did not adequately represent the repeats found in the *P. westermani* assembly, consistent with the distant evolutionary relationship of lung flukes with previously sequenced worm genomes. We therefore carried out *de-novo* repeat characterization using the RepeatModeller package, version 1.0.9 (RepeatModeler, RRID:SCR_015027), and used the generated consensus sequences to identify repetitive regions by RepeatMasker (RepeatMasker , RRID:SCR_012954), version 4.0.7 (both available at http://www.repeatmasker.org). To enable direct comparison with related trematode species we also ran RepeatModeller and RepeatMasker separately on the *F. hepatica*, *O. viverrini* and *C. sinensis* genomes with the same program parameters as those used for *P. westermani*.

A relatively high percentage (45.2%) of the *P. westermani* genome sequence was repeat-derived, similar to the rate reported for *Schistosoma* spp. (40.1-47.5%) (20-22) and *F. hepatica* (57.1%), but considerably higher than the rate observed for the closer relatives *O.*

viverrini (28.9%) and *C. sinensis* (32.6%) (**Table 3**). Retrotransposons of the long interspersed nuclear element (LINE) subtype were found to be the greatest contributors of repetitive DNA (21.6%) (**Table 3**), consistent with reports for other trematode genomes (23). In *P. westermani* and *F. hepatica*, the two largest of the four included trematode genomes, long terminal repeat (LTR) retrotransposons were also highly abundant contributing 7.7% and 10.1% of the genomes, respectively. Additionally, all four genomes had considerable amounts of repetitive DNA (10.7-17.1%) that did not match repeat consensus sequences of any of the known repeat classes modelled by RepeatModeler. The relatively high proportion of repeat-derived sequences in *P. westermani* may explain some of the increased size observed for this genome compared to the genomes of related flatworm species.

Table 3. Repeat content percentage of *P. westermani* and related trematode genome sequences.

Repeat class	*P. westermani*	*F. hepatica*	*O. viverrini*	*C. sinensis*
LINE	21.57	26.17	12.76	14.85
LTR	7.71	10.06	2.82	1.97
DNA elements	1.76	2.14	0.94	1.04
SINE	0.96	1.06	1.26	1.22
Simple repeats	0.18	0.63	0.43	0.36
Unclassified	12.97	17.06	10.69	13.15
**Total**	**45.15**	**57.12**	**28.9**	**32.59**

Gene prediction and functional annotation

Genes were predicted by the Maker pipeline, version 2.31.9, using Augustus (28), version 3.2.3, and GeneMark-ES (29), version 4.32, for *ab-initio* gene prediction. To accurately model the sequence properties of the *P. westermani* genome, both gene finders were initially trained by BRAKER1 (30), version 1.9, which makes use of mapped transcriptome sequence data. Previously published RNA-seq data from adult *P. westermani* (9) were obtained from the short read archive and mapped to our genome assembly using the Star aligner (31), version 2.5, with the option --twopassMode Basic. BRAKER1 was then run with default parameters. The RNA-seq data was further assembled into transcripts using cufflinks

(32), version 2.2.1, with the options --frag-bias-correct <p.westermani assembly> --multi-read-correct. The resulting transcripts were provided as input for Maker via the "est_gff" option. For homology based searches Maker was provided with the following wormbase v8 protein datasets: *Clonorchis sinensis* (PRJDA72781), *Opisthorchis viverrini* (PRJNA222628), *Schistosoma mansoni* (PRJEA36577), *Caenorhabditis elegans* (PRJNA13758), *Echinococcus granulosus* (PRJEB121), *Hymenolepis diminuta* (PRJEB507) and *Schistosoma haematobium* (PRJNA78265). Additionally, the Swiss-Prot dataset from UniProt was included. Maker was allowed to report single exon genes, and otherwise run with default parameters.

Proteins were functionally annotated based on a BLASTp search against the NCBI non-redundant protein database (obtained on 25.10.17) requiring an e-value <1e-15 and the best hit spanning at least 40% of the query sequence. KEGG annotations were identified using the BlastKoala server with the option "genus_eukaryotes" (33). Additionally, functional domains, GO annotations, transmembrane proteins and signal peptides were identified with InterProScan (InterProScan , RRID:SCR_005829)(34), version 5.25-64.0. GO annotations were then visualized using WEGO (35). In total, 12,852 protein encoding genes were predicted in the *P. westermani* genome and functionally annotated (**Table 2**).

Genome comparison

Predicted *P. westermani* coding genes were mapped to the genomes of related trematode species using Exonerate, version 2.4.0, requiring a minimal sequence identity of 30% and excluding matches spanning less than 40% of the query protein. The majority of predicted proteins (86.2%) had inferred homologs in the related trematode species (**Figure 3A**) and showed a similar distribution of protein functional categories (**Figure 3C**). The *P. westermani* predicted proteome was most similar to *O. viverrini* and *C. sinensis*. Of the 12,852 predicted proteins, 10,350 (80%) had inferred homologs in *O. viverrini* with an average sequence identity of 64.1%, and 10,227 (79.6%) had homologs in *C. sinensis* with an average sequence identity of 63.8% (**Figures 3A and 3B**).

Phylogenetic analysis and estimation of divergence time

A protein-based phylogenetic tree was inferred from 14 worm genomes, including *P. westermani,* 12 related trematode/cestode species and *Schmidtea mediterranea,* a free-living turbellarian flatworm, as outgroup (**Figure 4**). We first identified single-copy proteins shared across all 14 included worm species. Single-copy proteins were identified based on BLASTp searches of a species proteins against the species own proteome using a sequence-identity cut-off of 30% and requiring hits to cover >50% of the query sequence. Single-copy proteins shared across all 14 species were then identified using a less stringent BLASTp search with a 30% sequence identity cut-off but requiring only >40% coverage of the query sequence. We identified 104 single-copy proteins shared across the 14 worm species that were then aligned using MUSCLE (36). The resulting multiple sequence alignment was de-gapped with trimAI (37) and a phylogenetic tree was reconstructed by PhyML (PhyML , RRID:SCR_014629)(38). Model selection in PhyML (39) identified the LG model (40) with decorations +G+I+F as optimal. PHYLIP v3.696 (41) using the maximum likelihood method and the Jones-Taylor-Thornton (JTT) probability model (42) resulted in the same tree topology, demonstrating the robustness of the inferred phylogenetic relationships.

The multiple alignment and the inferred phylogenetic tree were then used to estimate species divergence by a Bayesian model with relaxed molecular clock using MCMCTREE in PAML 4.9e (**Figure 4**)(PAML , RRID:SCR_014932). The model was calibrated based on previously published divergence times and ages of fossil records. Evidence for trematode infestation have been reported from the Eocene (56 to 33.9 million years (myr) ago) and preserved trematode eggs have been found in dinosaur coprolites from the Early Cretaceous (146 to 100 myr ago); however, fossil records indicate that trematodes may have already existed more than 400 myr ago (43, 44). The trematode split from other neodermatan lineages was therefore fixed at >56 myr. The origin of schistosomes has been estimated somewhere in the Miocene around 15-20 myr ago (45, 46). It has further been estimated that the divergence of *S. mansoni* did likely not occur before 2-5 myr ago, based on fossil records of its intermediate host *Biomphalaria* (47). From these data, the split of Plagiorchiida (including *P. westermani*) and Opistorchiida (including *O. viverrini* and *C. sinensis*) was estimated to have occurred 38.9 myr ago (95% confidence interval of 28.0-58.6 myr) (**Figure 4**). To estimate the robustness of the inferred divergence times the analysis was repeated using BEAST 2 version 2.5.0 (48), based on the JTT substitution matrix,

gamma category count of 4, estimated substitution rate, relaxed clock log normal model, and a chain length of 6M (49, 50). A maximum clade credibility tree using median node heights was generated by the BEAST 2 treeannotator tool. Divergence times inferred by BEAST 2 well matched the MCMCTREE results and were within the estimated confidence intervals (Figure 4). The split of the Plagiorchiida and the Opistorchiida was estimated to have occurred 31.5 myr ago.

Discussion

We have presented the first whole-genome sequence of a *Paragonimus* spp. worm, providing a valuable resource to the field that will aid our understanding of this group of clinically important parasites. The genome was found to be unusually large for a worm, a feature that at least in part appears attributable to an expansion of retrotransposable elements, rather than genome duplication events.

The mitochondrial genome was also found to be very large comprising 20.3 Kb. Such a large size appears to be a common feature of worms and results from a long repetitive region of unknown function. However, while this region appears to be a feature of most flatworms it is rarely sequenced in full due to the technical challenges of sequencing long tandemly repeated sequences.

P. westermani has been described as a species complex with considerable genetic differences across geographic regions (2). The genome presented herein is of an Indian isolate and it will be of considerable interest to compare this and the genomes of isolates from other regions where *P. westermani* is endemic to elucidate the region specific genetic features. This would be particularly informative as not all endemic regions are associated with paragonimiasis in humans (2).

Phylogenetic analyses of *P. westermani* shows that it has diverged considerably from its closest relatives, *Clonorchis sinensis* and *Opisthorchis viverrini* with a split estimated to have occurred 28-59 myr ago. Subsequent to that split the species spread out across a vast geographical range, acquiring distinct local traits in what may eventually be considered speciation events. This time-span has also seen an expansion of two repeat families, in

particular the LINE and LTR elements. In mammals these elements are known to occasionally become exapted and gain novel regulatory functions (51), and they are therefore likely to add to the diversity of the *P. westermani* species complex.

Conclusion

The presented *P. westermani* genome assembly provides new insights into the molecular biology of *Paragonimus* and provides an unprecedented resource for functional studies of lung flukes and for the design of new disease interventions and diagnostics tests.

Availability of supporting data

The nuclear and mitochondrial genomes are available from NCBI under accession number PRJNA454344. Annotation and tree data is available from the *GigaScience* GigaDB repository (52).

Abbreviations

bp base pair

BUSCO Benchmarking Universal Single-Copy Orthologs

Kb Kilo base pair

LNR Long noncoding region

LINE Long interspersed nuclear elements

LTR Long terminal repeat

Mb Mega base pair

MYR Million years

SINE Short interspersed nuclear elements

Competing interests

All authors declare that they have no competing interests.

Funding

This work has been supported by grants from the QIMR Berghofer Medical Research Institute (Chenhall Estate) and the Australian Infectious Diseases Research Centre. DPM is a National Health and Medical Research Council Senior Principal Research Fellow.

Author contributions

LK and DPM conceived and managed the project; KN and KRD provided *P. westermani* material. TA and SN isolated genomic DNA. MZ and GG managed DNA sequencing. HO carried out genome assembly, gene prediction and functional genome annotation. HO and LK carried out comparative genomics. LK, DPM, MKJ and MAR attracted funding and designed the study. LK and HO drafted the manuscript and all authors read, edited and approved the final manuscript.

Acknowledgements

We wish to acknowledge the QIMR Berghofer Medical Research Institute (Chenhall Estate donation) and the Australian Infectious Diseases Research Centre for funding this work.

References

1. Furst T, Keiser J, Utzinger J. Global burden of human food-borne trematodiasis: a systematic review and meta-analysis. Lancet Infect Dis. 2012;12(3):210-21.
2. Blair D. Paragonimiasis. Adv Exp Med Biol. 2014;766:115-52.
3. Roy JS, Das PP, Borah AK, Das JK. Paragonimiasis in a Child from Assam, India. J Clin Diagn Res. 2016;10(4):DD06-7.
4. Singh TS, Hiromu S, Devi KR, Singh WA. First case of Paragonimus westermani infection in a female patient in India. Indian J Med Microbiol. 2015;33 Suppl:156-9.
5. Jones MK, Keiser J, McManus DP. Trematodes. In: Jorgensen JH, Pfaller MA, Carroll KC, Funke G, Landry ML, Richter SS, et al., editors. Manual of Clinical Microbiology, Eleventh Edition: American Society of Microbiology; 2015.
6. Luo J, Wang MY, Liu D, Zhu H, Yang S, Liang BM, et al. Pulmonary Paragonimiasis Mimicking Tuberculous Pleuritis: A Case Report. Medicine (Baltimore). 2016;95(15):e3436.
7. Zhou R, Zhang M, Cheng N, Zhou Y. Paragonimiasis mimicking chest cancer and abdominal wall metastaisis: A case report. Oncol Lett. 2016;11(6):3769-71.
8. Kalhan S, Sharma P, Sharma S, Kakria N, Dudani S, Gupta A. Paragonimus westermani infection in lung: A confounding diagnostic entity. Lung India. 2015;32(3):265-7.
9. Li BW, McNulty SN, Rosa BA, Tyagi R, Zeng QR, Gu KZ, et al. Conservation and diversification of the transcriptomes of adult Paragonimus westermani and P. skrjabini. Parasit Vectors. 2016;9:497.
10. Marcais G, Kingsford C. A fast, lock-free approach for efficient parallel counting of occurrences of k-mers. Bioinformatics. 2011;27(6):764-70.
11. Song L, Bian C, Luo Y, Wang L, You X, Li J, et al. Draft genome of the Chinese mitten crab, Eriocheir sinensis. Gigascience. 2016;5:5.
12. Hackl T, Hedrich R, Schultz J, Forster F. proovread: large-scale high-accuracy PacBio correction through iterative short read consensus. Bioinformatics. 2014;30(21):3004-11.
13. Chevreux B, Pfisterer T, Drescher B, Driesel AJ, Muller WE, Wetter T, et al. Using the miraEST assembler for reliable and automated mRNA transcript assembly and SNP detection in sequenced ESTs. Genome research. 2004;14(6):1147-59.
14. Luo R, Liu B, Xie Y, Li Z, Huang W, Yuan J, et al. SOAPdenovo2: an empirically improved memory-efficient short-read de novo assembler. Gigascience. 2012;1(1):18.
15. Simpson JT, Wong K, Jackman SD, Schein JE, Jones SJ, Birol I. ABySS: a parallel assembler for short read sequence data. Genome research. 2009;19(6):1117-23.
16. Boetzer M, Henkel CV, Jansen HJ, Butler D, Pirovano W. Scaffolding pre-assembled contigs using SSPACE. Bioinformatics. 2011;27(4):578-9.
17. Hunt M, Kikuchi T, Sanders M, Newbold C, Berriman M, Otto TD. REAPR: a universal tool for genome assembly evaluation. Genome Biol. 2013;14(5):R47.
18. Wang X, Chen W, Huang Y, Sun J, Men J, Liu H, et al. The draft genome of the carcinogenic human liver fluke Clonorchis sinensis. Genome Biol. 2011;12(10):R107.
19. Young ND, Nagarajan N, Lin SJ, Korhonen PK, Jex AR, Hall RS, et al. The Opisthorchis viverrini genome provides insights into life in the bile duct. Nat Commun. 2014;5:4378.
20. Young ND, Jex AR, Li B, Liu S, Yang L, Xiong Z, et al. Whole-genome sequence of Schistosoma haematobium. Nat Genet. 2012;44(2):221-5.
21. Berriman M, Haas BJ, LoVerde PT, Wilson RA, Dillon GP, Cerqueira GC, et al. The genome of the blood fluke Schistosoma mansoni. Nature. 2009;460(7253):352-8.
22. Schistosoma japonicum Genome S, Functional Analysis C. The Schistosoma japonicum genome reveals features of host-parasite interplay. Nature. 2009;460(7253):345-51.
23. Cwiklinski K, Dalton JP, Dufresne PJ, La Course J, Williams DJ, Hodgkinson J, et al. The Fasciola hepatica genome: gene duplication and polymorphism reveals adaptation to the host environment and the capacity for rapid evolution. Genome Biol. 2015;16:71.

24. Simao FA, Waterhouse RM, Ioannidis P, Kriventseva EV, Zdobnov EM. BUSCO: assessing genome assembly and annotation completeness with single-copy orthologs. Bioinformatics. 2015;31(19):3210-2.
25. Bernt M, Donath A, Juhling F, Externbrink F, Florentz C, Fritzsch G, et al. MITOS: improved de novo metazoan mitochondrial genome annotation. Mol Phylogenet Evol. 2013;69(2):313-9.
26. Laslett D, Canback B. ARAGORN, a program to detect tRNA genes and tmRNA genes in nucleotide sequences. Nucleic Acids Res. 2004;32(1):11-6.
27. Le TH, Blair D, McManus DP. Mitochondrial genomes of parasitic flatworms. Trends Parasitol. 2002;18(5):206-13.
28. Stanke M, Steinkamp R, Waack S, Morgenstern B. AUGUSTUS: a web server for gene finding in eukaryotes. Nucleic Acids Res. 2004;32(Web Server issue):W309-12.
29. Lomsadze A, Ter-Hovhannisyan V, Chernoff YO, Borodovsky M. Gene identification in novel eukaryotic genomes by self-training algorithm. Nucleic Acids Res. 2005;33(20):6494-506.
30. Hoff KJ, Lange S, Lomsadze A, Borodovsky M, Stanke M. BRAKER1: Unsupervised RNA-Seq-Based Genome Annotation with GeneMark-ET and AUGUSTUS. Bioinformatics. 2016;32(5):767-9.
31. Dobin A, Davis CA, Schlesinger F, Drenkow J, Zaleski C, Jha S, et al. STAR: ultrafast universal RNA-seq aligner. Bioinformatics. 2013;29(1):15-21.
32. Trapnell C, Williams BA, Pertea G, Mortazavi A, Kwan G, van Baren MJ, et al. Transcript assembly and quantification by RNA-Seq reveals unannotated transcripts and isoform switching during cell differentiation. Nat Biotechnol. 2010;28(5):511-5.
33. Kanehisa M, Sato Y, Morishima K. BlastKOALA and GhostKOALA: KEGG Tools for Functional Characterization of Genome and Metagenome Sequences. J Mol Biol. 2016;428(4):726-31.
34. Jones P, Binns D, Chang HY, Fraser M, Li W, McAnulla C, et al. InterProScan 5: genome-scale protein function classification. Bioinformatics. 2014;30(9):1236-40.
35. Ye J, Fang L, Zheng H, Zhang Y, Chen J, Zhang Z, et al. WEGO: a web tool for plotting GO annotations. Nucleic Acids Res. 2006;34(Web Server issue):W293-7.
36. Edgar RC. MUSCLE: multiple sequence alignment with high accuracy and high throughput. Nucleic Acids Res. 2004;32(5):1792-7.
37. Capella-Gutierrez S, Silla-Martinez JM, Gabaldon T. trimAl: a tool for automated alignment trimming in large-scale phylogenetic analyses. Bioinformatics. 2009;25(15):1972-3.
38. Guindon S, Dufayard JF, Lefort V, Anisimova M, Hordijk W, Gascuel O. New Algorithms and Methods to Estimate Maximum-Likelihood Phylogenies: Assessing the Performance of PhyML 3.0. Systematic Biology. 2010;59(3):307-21.
39. Lefort V, Longueville JE, Gascuel O. SMS: Smart Model Selection in PhyML. Mol Biol Evol. 2017;34(9):2422-4.
40. Le SQ, Gascuel O. An improved general amino acid replacement matrix. Mol Biol Evol. 2008;25(7):1307-20.
41. Felsenstein J. Confidence-Limits on Phylogenies - an Approach Using the Bootstrap. Evolution. 1985;39(4):783-91.
42. Jones DT, Taylor WR, Thornton JM. The Rapid Generation of Mutation Data Matrices from Protein Sequences. Computer Applications in the Biosciences. 1992;8(3):275-82.
43. Poinar G, Jr., Boucot AJ. Evidence of intestinal parasites of dinosaurs. Parasitology. 2006;133(Pt 2):245-9.
44. Huntley JW, De Baets K. Trace Fossil Evidence of Trematode-Bivalve Parasite-Host Interactions in Deep Time. Adv Parasitol. 2015;90:201-31.
45. Littlewood DTJe, Baets Kde. Fossil parasites.
46. Snyder SD, Loker ES. Evolutionary relationships among the Schistosomatidae (Platyhelminthes:Digenea) and an Asian origin for Schistosoma. J Parasitol. 2000;86(2):283-8.
47. Morgan JA, Dejong RJ, Snyder SD, Mkoji GM, Loker ES. Schistosoma mansoni and Biomphalaria: past history and future trends. Parasitology. 2001;123 Suppl:S211-28.

48. Bouckaert R, Heled J, Kuhnert D, Vaughan T, Wu CH, Xie D, et al. BEAST 2: A Software Platform for Bayesian Evolutionary Analysis. Plos Computational Biology. 2014;10(4).
49. Heled J, Drummond AJ. Calibrated tree priors for relaxed phylogenetics and divergence time estimation. Syst Biol. 2012;61(1):138-49.
50. Drummond AJ, Ho SY, Phillips MJ, Rambaut A. Relaxed phylogenetics and dating with confidence. PLoS Biol. 2006;4(5):e88.
51. Mita P, Boeke JD. How retrotransposons shape genome regulation. Curr Opin Genet Dev. 2016;37:90-100.
52. Oey H; Zakrzewski M; Narain K; Devi KR; Agatsuma T; Nawaratna S; Gobart G; Jones M; Ragan MA; McManus DP; Krause L (2018): Supporting data for "Complete genome sequence of the oriental lung fluke Paragonimus westermani" GigaScience Database. http://dx.doi.org/10.5524/100524

Figure legends:

Figure 1. K-mer frequencies for the 450bp library. Distribution of 17-mers in the 450bp short-insert library demonstrated low sequence heterozygosity. We observed a single peak at 26x and the *P. westermani* genome size was estimated to be 1.1 Gb.

Figure 2. The complete *P. westermani* mitochondrial genome.

A graphical representation of the *P. westermani* circular mitochondrial genome is shown, including a ~6.9 Kb repetitive region. Three distinct repeat units were identified in this region, as well as an intervening tRNA gene (tRNA-Glu). All genes are transcribed in the clock-wise direction.

Figure 3. Conservation of the *P. westermani* proteome across four related trematode species. *P. westermani* proteins were mapped to the genome sequences of *O. viverrini, C. sinensis, F. hepatica* and *S. mansoni* using Exonerate. A) *P. westermani* centred Venn diagram of 12,852 predicted proteins. The four included trematode species shared a core set of 7,599 proteins. B) Sequence identity of *P. westermani* proteins and orthologues inferred in genomes of related trematodes. Average sequence identity is given in brackets. C) Distribution of identified functional GO categories across three trematode species. GO annotations were assigned by InterProScan and visualized using WEGO.

Figure 4. Phylogenetic tree and estimated divergence times. A phylogenetic tree of selected trematodes and cestodes and *S. mediterranea* as outgroup was reconstructed from 104 shared single-copy proteins using the maximum likelihood method. Species divergence was estimated by a Bayesian model using MCMCTREE with relaxed molecular clock and is given in million years with 95% confidence intervals in round brackets. The split of *P. westermani* was estimated to have occurred somewhere around 38.9 myr ago (28.0-58.6 myr). The analysis was repeated using BEAST 2 and estimated divergence times are shown in square brackets. BEAST 2 estimated the split of *P. westermani* to have occurred 31.5 myr ago.

 Click here to access/download;Figure;Figure 1.png

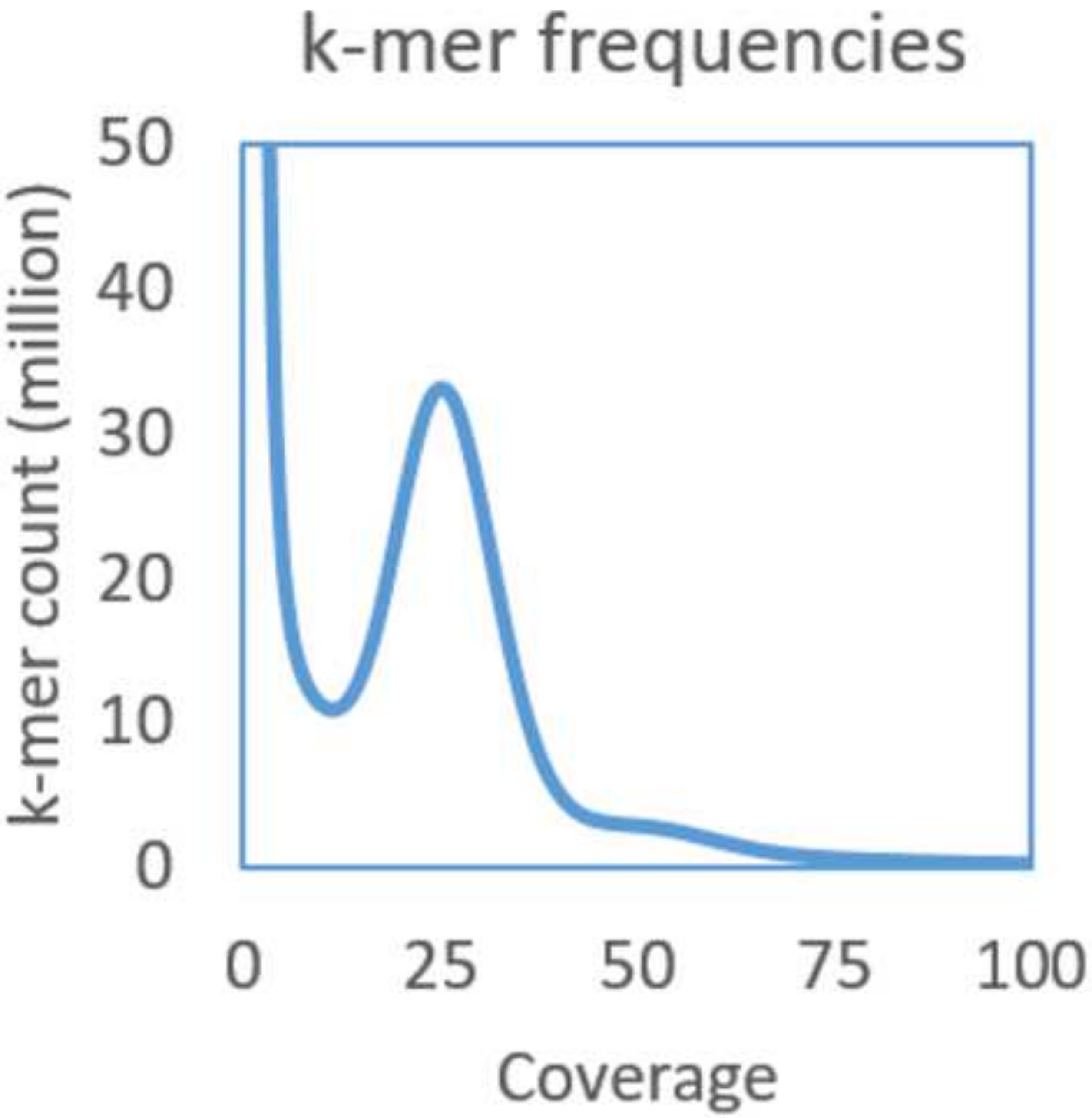

Figure2

Click here to access/download;Figure;Figure 2.jpg

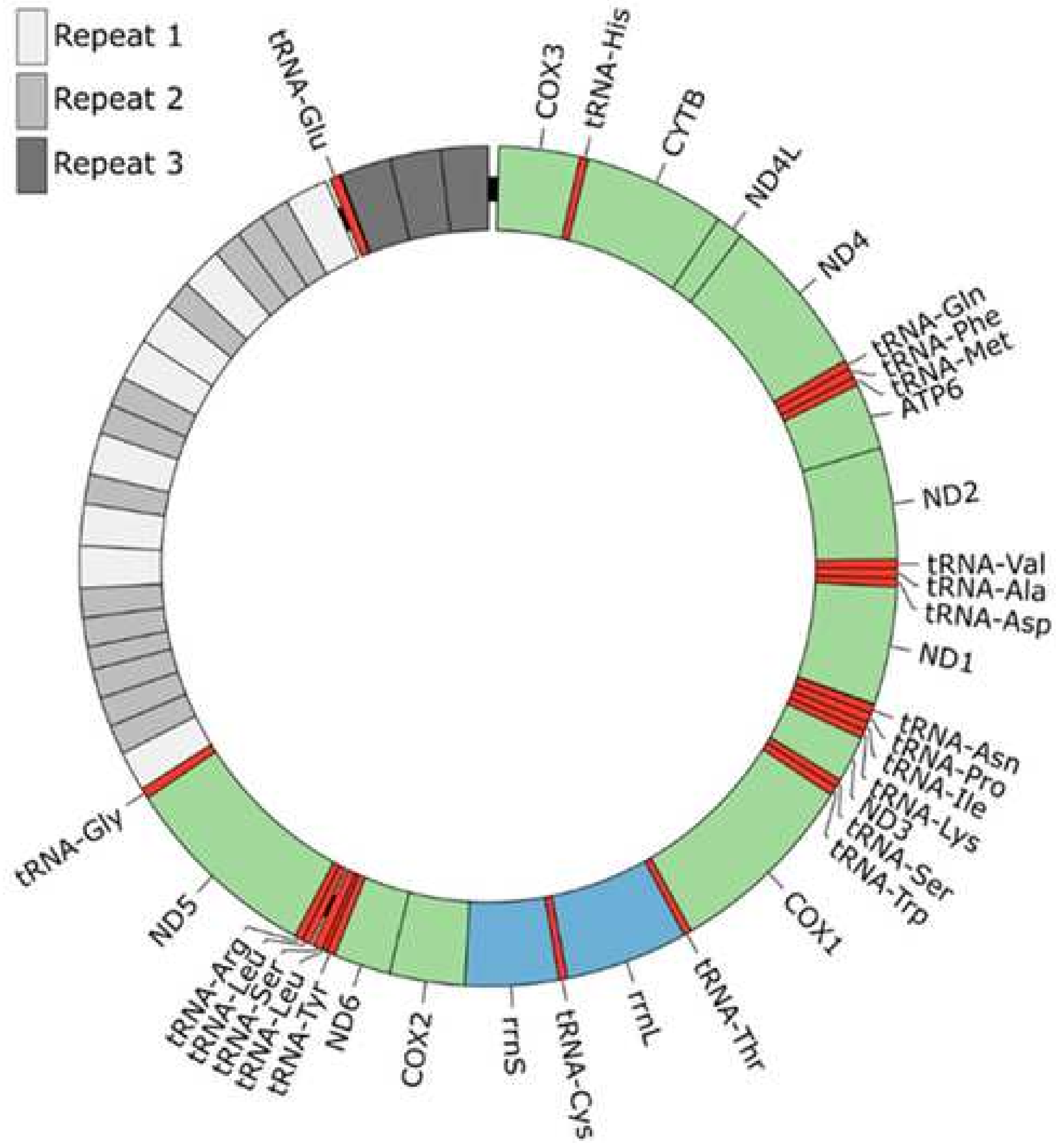

Figure3

Click here to access/download;Figure;Figure 3.png

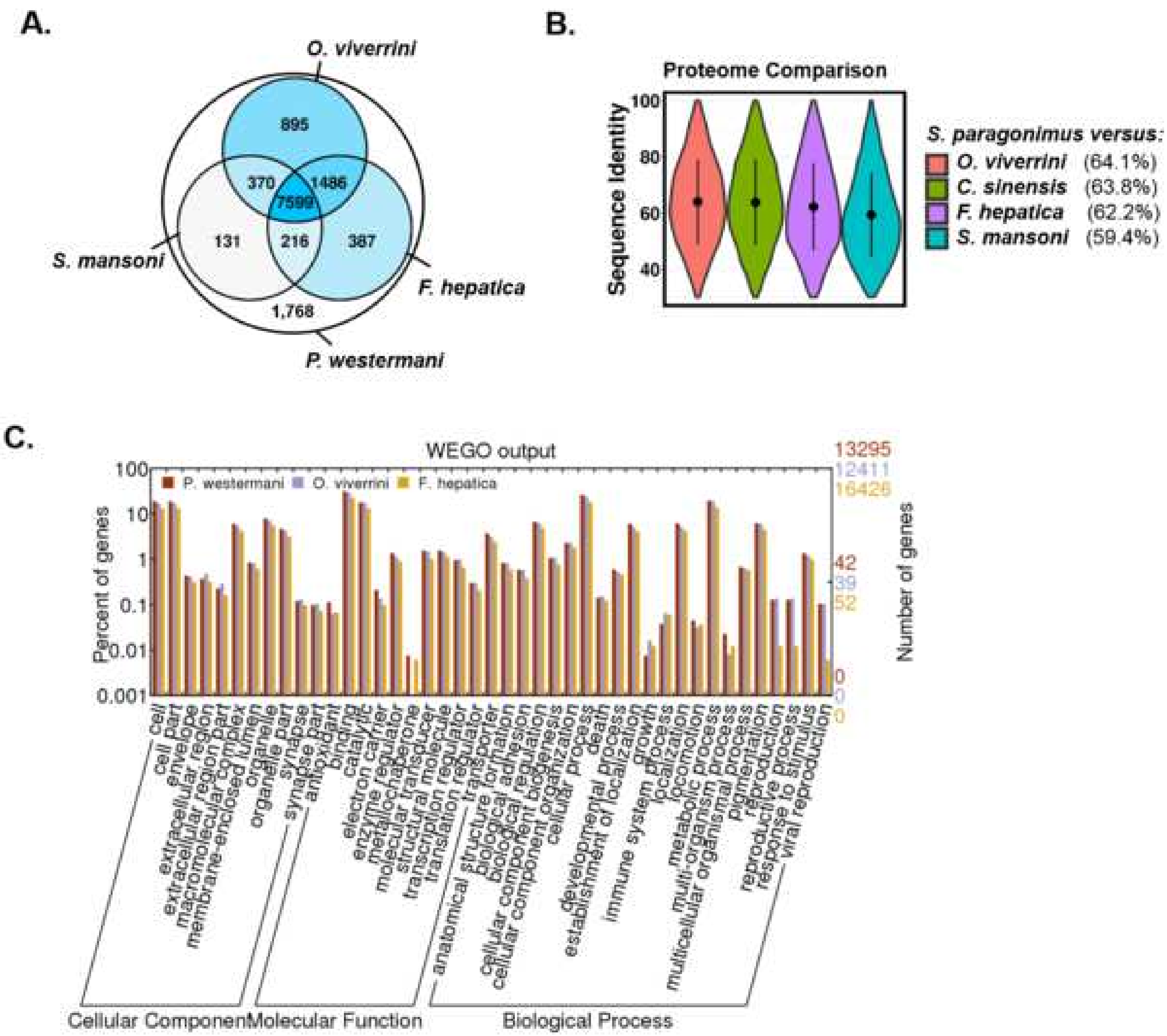

Figure4

Click here to access/download;Figure;Figure 4.png

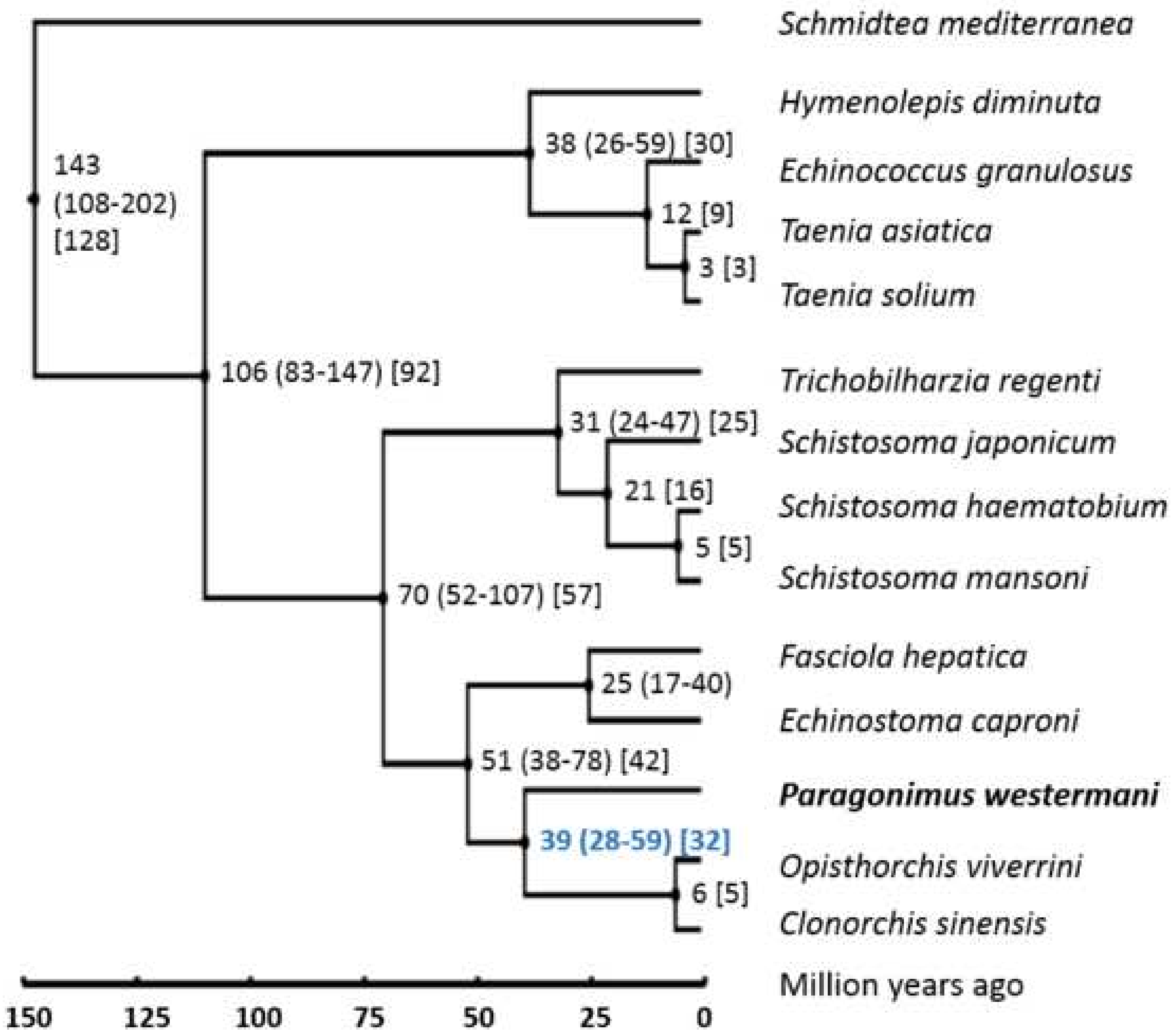

Additional File 1

Click here to access/download
Supplementary Material
Additional file 1.pdf

Personal Cover

Click here to access/download;Personal Cover;CoverLetter.docx

Dear Scott Edmunds (Executive Editor),

Thank you for considering our manuscript “Complete genome sequence of the oriental lung fluke *Paragonimus westermani*” (GIGA-D-18-00193) for publication in GigaScience.

The reviewers were generally positive in their comments about the manuscript. However they did raise valuable points that we have addressed. We have prepared a detailed response to the points. Furthermore, we have modified sections of the manuscript and figures to address reviewer’s questions. The changes have been highlighted (yellow) in the main text of the manuscript.

Both reviewers questioned the manuscript type. We have resubmitted the manuscript as a Data Note.

Yours sincerely,

Lutz Krause, PhD
Principal Research Fellow / Associate Professor
Head, Computational Medical Genomics Group
The University of Queensland Diamantina Institute
