# Supplementary material for: Whole-genome sequence of the oriental lung fluke Paragonimus westermani
Source: Gigascience. 2018 Dec 6;8(1):giy146. doi: 10.1093/gigascience/giy146 (PMC6329441; doi:10.1093/gigascience/giy146)
Supplement: Supplemental File [file giy146_supplemental_file.pdf]

Repeat1 (328bp ~8 copies)
TGTCAAGTTTGAAGGGACCGATTTAGCTTCGATTCCAATGGGTGTAGAGGTTTGGAGTTGGC
GTTGCCTGTTGATTTTCTGTGTCAAGGGGGGTCTGAAACTATGCGCGAAAAGGGTGCAAAAA
AAATTCGTAAGGGGGGGGCATTGCAAACTTTTTCCTTTTTAAAAATTTACAGCTTAATTCAG
GTCTAGTCGAAGAGTGAAGTGGTTTTTATCTCCCCTTAATTTGACTGTCGATTAAAAATTTT
CGTTACTTTTGTGTCAAAATTACATCATAGCTTTTTTCAGGGGAGTTCGGAGGTGAAAAGTT
GGATTTTTGAAGGGTTTG

Repeat2 (229bp ~13 copies)
TGTCAAGTTTGAAGGGACCGATTTGGCTTCGATTCCAATGGGTGTAGAGGTTTGGAGTTGGC
GTTGCCTGTTGATTTTCTGTGTCAAGGGGGGTTTTAAACTATGCTGTCGAGGGTGTTCACCG
TAGCTTTTTTCAGGGGAGTTCGGAGGTGAAAAGTTCGGTTTTTTCGATGAGCTGGTACGAAA
TGCTATTATGTTAATCATAAGTAGAGTTATAATTAGAGGTCTC

Repeat3 (406bp 3 copies)
AAAAAAAGATATCATCGCTAAAAGAGAATAATTGGAAATGACTGTGTTGTCGTAAAGGATTG
GGATTAACTGTAGTCCGAGCAGTGTTGGTTGTGCGAGGAAGAGATGAGGGGGGCTTAATAAT
AATATGAGGAGGCCCCTACAATGAAAGTGGTTAAGAAGGTCCATTGTAAAGGATTAGGATTA
ACTGTAGTCCGAGCAGTGTTGGTTGTGCGAGGAAGAGATGAGGGGGGCTTAATAATAATATG
AGGAGGCCCCTACAATGAAAGTGGTTAAGAAGGTCCATTGTAAAGGATTAGGATTAACTGTA
GTCCGAGCAGTGTTGGTTGTGCGAGGAAGAGATGAGGGGATCTTTGGTTAATAATACAGGAA
GTCATCTGTAATGGGGGGAAAGGGGGGCCTGACC

Additional File 1: Consensus sequences for three distinct repeat units in the *P. Westermani* mitochondrial genome
