# Supplementary material for: Whole-genome sequence of the oriental lung fluke Paragonimus westermani
Source: Gigascience. 2018 Dec 6;8(1):giy146. doi: 10.1093/gigascience/giy146 (PMC6329441; doi:10.1093/gigascience/giy146)
Supplement: Response_to_Reviewer_Comments_Original_Submission.pdf [file giy146_response_to_reviewer_comments_original_submission.pdf]

Author's Response To Reviewer Comments

Close

Reviewer: 1
1 – I suggest a small change in the manuscript title: "Draft Whole genome sequence of the oriental lung fluke…" or just "Whole genome sequence of the …". The term complete for nuclear genome sequence means that it is the final version (in chromosome level with no gaps), not the case here where the genome is still in 30,977 pieces, so complete should be not used here. The mitochondrial indeed looks complete.
Response: We have changed the manuscript title to "Whole-genome sequence of the oriental lung fluke Paragonimus westermani" as suggested by the reviewer.

2 – The authors did not mention how they removed potential contamination or how they maintained the pathogen for the DNA extraction (Please add this information)
Response: Comparison of assembled scaffolds with public genome sequence data identified contamination by rat (the experimental host) and the bacterium Delftia sp. All sequences mapped to these genomes were removed. We have now added this information to the methods section.

3 – Table 1 could be used as supplemental material
Response: We intend to submit the manuscript as Data Note. We believe that Table 1 is important for a Data Note and suggest we keep it in the main manuscript.

4 – The assembly was performed by well-known genome assemblers, but there was any particular reason to not use any of the two most used PacBio assemblers (HGAP and CANU?)
Response: We have used CANU for several parasite genomes. The program worked well for other parasites (manuscript in review), but did not perform well on this particular genome. Mira worked better for Paragonimus and generated a single complete mitochondrial contig, whereas CANU resulted in multiple shorter contigs.

5 – The authors choose to use for the Illumina assembly the ABYSS assembler. From my personal experience and from some colleagues there are several other assemblers that give a better job than ABYSS (Spades, MIRA, Velvet and SoapDenovo2). I know that it varies depending of the nature of the organism and sample used for the assay, but since the group used for the gapfilling step the soapDenovo gapcloser, I would like to see in the manuscript some information about why these pipelines were choosen beside others
Response: We have evaluated several assembly programs and ABYSS performed best for this particular genome. ABYSS is also one of the few assemblers that allow inclusion of long-read data to guide scaffolding. The program is still widely used and well maintained. We have an established pipeline using SoapDenovo2, which has been used for the assembly of other parasite genomes (manuscript in review). However, SoapDenovo2 did not perform well for this particular genome, with a large size and many repetitive regions. Additionally, the Paragonimus genome was sequenced from 50 individual worms, resulting in a low-level sequence heterogeneity and assembly of this data proofed to be challenging. ABYSS performed particularly well for the assembly of contigs for this genome. However, the ABYSS gap filler is not well suited for closing gaps larger than 1kb (according to the

ABYSS manual and our own experience), whereas the soapDeNovo gapfiller is well suited for this task and performed particularly well on this genome. Additional information has been added to the methods section.

6 – Line 179 - REAPR typo. I would also suggest the authors to perform for this final polishing genome correction step Pilon or ICORN2 using the Illumina reads generated
Response: We thank the reviewer for this suggestion. However, Pilon does not seem to perform well for this particular genome. Genome polishing using Pilon with a variety of different settings actually resulted in a slight reduction of BUSCO scores (original assembly: 65.3% complete proteins; after Pilon: 63.9% complete proteins), indicating that Pilon did not improve the overall quality of this particular genome assembly. We manually investigated Pilon results and postulate that Pilon was misled by low-level sequence heterogeneity caused by the pooling of 50 individual worms. As the genome has already been deposited in NCBI and passed all manual QC checks we believe that the questionable improvements by Pilon do not justify re-submission of an updated genome to NCBI.

7 – Please add more information about the genome assembly statistics in table 2 (L50 and number of Ns), a quick run on QUAST should give you this information. And please explain if these gaps are just generated during the scaffolding by the mate pair evidence or it was also generated for unknown size gaps (100Ns). This information is really important to show that some regions could be missing in this draft genome assembly, so future studies could be aware of this fact;
Response: We have run the assembly through QUAST, as requested, and added the L50 to Table 2. The number of Ns can already be inferred from Table 2 as we provide the size of the genome both with and without counting Ns (“Assembly size” and “Total base pairs”). We have re-named “Total base pairs” to “Ungapped size” to make this clearer. The Gaps are generated both during contig assembly (abyss) and scaffolding (SSPACE) and represent the estimated size of the gaps. We have added a sentence to the manuscript to make this clear.

8 – Line 250 - Since the ncRNA information was so important in the mitochondrial annotation, and the group already characterized the tRNAs, please add the method to predict these tRNAs (like tRNAscan) and also, I suggest adding an Aragorn or inferno ncRNA prediction run to improve even more the annotation
Response: The program Mitos, which was used to characterize the mitochondrial genome, identifies both non-coding RNAs and proteins. However, Aragorn was also run to identify any additional tRNAs in the mitochondrial genome (added to methods).

9 – Line 258 - no problem with the methodology, but Cufflinks has a substitute, StringTie (Petera at el., 2015). It will do a much better job to assembly the transcriptome
Response: StringTie was not available when the project started, but we thank the reviewer for this suggestion and will evaluate StringTie for future projects. Cufflinks is well established (>5,000 citations), proven to generate accurate results and is still widely used. We agree that there are many alternative tools that could have been used for transcriptome assembly, but our group has an established and well tested pipeline using cufflinks. We have extensive experience with cufflinks and have optimized the parameters to generate robust and high-quality results. We would further like to point out that we don’t publish the assembled cDNA data.

10 – Genome Comparison - I understand that this was not the focus of this manuscript, but sequence identity besides important is a too general comparison method. I suggest add a orthology analysis and maybe generate a Circos synteny plot comparing the new genome with the most similar species available
Response: We will submit the manuscript as Data Note and therefore believe that additional comparative analysis are not required.

11 – Phylogeny - Add a Modeltest run to check if Jones-Taylor-Thornton (JTT) was the best substitution method to be used. For the ML analysis I suggest using PhyML instead of Phylip again, the software used is good but better and newer ones were developed
Response: As suggested, we have now repeated the phylogenetic analysis using PhyML and a model test found the LG substitution model with decorations +G+I+F as optimal. The JTT model was the second best model. PhyML using the LG+G+I+F model resulted in exactly the same tree topology as our previous analysis using Phylip with the JTT model, demonstrating the robustness of our inferred phylogenetic relationships.

12 – Bayesian method - MCMCTREE in PAML is good, but since Bayesian methods tend to vary, I suggest the group to run another test using the most known softwares (BEAST or mrBayes), to check if these mrca inferences are matching properly
Response: As suggested, we have now estimated divergence times using BEAST version 2. BEAST v2 estimates matched our previous results from MCMCTREE well and were within the estimated confidence intervals. Divergence times estimated by BEAST v2 were added to Figure 4 of the manuscript.

13 – Figure 1 - Doesn't need to be a main figure. Could be used as supplementary figure.
Response: The manuscript has been changed to Data Note and we believe that Figure 1 is important for this manuscript type.

14 – Figure 2 B - These sequences could be mentioned in the text and added as supplementary file. You can name these repeats if needed in figure 2 A.
Response: We agree and have moved the text to the supplementary data.

15 – Figure 3 - Figure is fine but needs to improve image quality. It is preferable to have a Venn diagram of the orthologs between these species.
Response: We have now replaced the figure with a non-proportional Venn diagram.

16 – Add a circus synteny plot figure between the new genome and the closest species genome available.
Response: We have re-submitted the manuscript as Data Note and we believe that in this case a synteny plot is not needed. Additionally, while we agree that a synteny plot would be valuable, generating a synteny plot would be problematic for the Paragonimus genome, as no close relative genome of high quality is available that would allow ordering of the scaffolds.

17 – Figure 4 - (optional) Try to make the same figure using Figtree. They have a nicer way to show the median of the mrca on each node.
Response: We have now improved the figure and aligned the numbers with the tree branches.

Reviewer 1 minor comments:
18 – Change the word faeces for stool. It's not wrong, but stool is more commonly used worldwide;
Response: We have changed “faeces” to “stool” as suggested.

19 – Line 148 - Data Sequencing: add the Illumina Platform used in the data generation (example: HiSeq2000)
Response: Done as suggested.

20 – Line 150 - Data Sequencing: add the PacBio Platform used in the data generation (example: PacBio Sequel or RSII)
Response: Done as suggested.

Reviewer: 2
The manuscript is currently written in my opinion as a data note rather than a research type manuscript. If this is the intention this should be made clearer by the authors as part of their submission. If the manuscript is intended to be submitted as a research paper, the authors should expand on their discussion and conclusions of their data.
Response: We have resubmitted the manuscript as a Data Note.

1 – Abstract, line 85 and Data description, line 157: The authors computationally determined the estimated size of the P.westermani genome, prior to assembly of the raw reads. The computationally determined estimated size was slightly larger than the assembled genome size. The authors should comment on the size difference. In addition, the authors interchange throughout the manuscript whether they compare the estimated size or the assembled genome size with other known published trematode genomes. Until it can be shown that the genome of P.westermani is actually 1.1 Gb, the authors should only refer to the assembled genome size particularly in the section around line 157, as these published trematode genomes describe only the assembled genome sizes.
Response: As suggested, we have added a comment regarding the genome size differences and now base the genome size comparison on the assembled genome sizes.

2 – Line 144 - at what point of infection were the parasites recovered - specifically how old were the parasites?
Response: The parasites were 30-40 days old, this information has been added to the manuscript.

3 – Lines 144-146 -Further information is required regarding the methodology of genomic

DNA extraction. Was the extraction carried out on individual worms and then combined or were the worms combined for extraction? Was the genomic DNA quality checked?
Response: The following information has been added to the manuscript: “Genomic DNA was isolated from a pool of 50 worms (30 – 40 days of age), yielding 18 µg of DNA. DNA was quantified by Pico green, QUBIT and NanoDrop. Degradation was tested by Microplate Reader and Agarose Gel Electrophorese (concentration of agarose gel: 1%, electrophoresis time: 40 min, voltage 150 V).

4 – Line 150 - can the authors confirm that the PacBio sequencing was performed on the same sample of genomic DNA?
Response: We confirm that the same sample of genomic DNA was used for PacBio and Illumina sequencing.

5 – Line 255 - the authors should mention that the RNAseq data was from adult parasites only, not the various different lifecycle stages.
Response: This information has now been added to the manuscript.

6 – Line 221 - Related to point 3, as the authors extracted DNA from 50 individual worms, did they check the level of polymorphism at the individual worm level for this region?
Response: We agree with reviewer that this would be an interesting question. However, DNA was isolated from a pool of 50 individual worms. Moreover, only 5 reads spanned the region in full (anchored in non-repetitive sequence at both ends), which was sufficient to generate a consensus sequence for the region, but not to accurately quantify individual-level differences.

7 – Line 272-273 - If the authors are submitting a research themed manuscript, they could include some further discussion of the predicted protein coding genes, particularly those predicted proteins that have inferred homologs in other trematodes (Fig 3A) and the Paragonimus-specific predicted proteins.
Response: The manuscript has been re-submitted as Data Note.

8 – Figure 3A - the venn diagram is currently difficult to interpret, particularly given its current small size as a multi-panel figure. I suggest amending this figure to a classical venn diagram or an Upset plot.
Response: The figure has been replaced by a non-proportional Venn diagram.

9 – The authors should include supplemental data detailing the functional annotation particularly the analysis of the functional domains, transmembrane domains and signal peptides, as well the data relating to the single copy predicted proteins used for the phylogenetic analysis.
Response: As requested, we have now uploaded our InterProScan results as well as the sequences for single copy proteins used for the phylogenetic analysis to the GigaScience ftp server.

10 – Minor corrections:
a. line 118 - develop into sporocysts
b. line 161 - 1.3 Gb
c. line 204, 291, 293 - BLAST
d. line 281 - predicted proteome
Response: We thank the reviewer for these comments. The manuscript has been modified as suggested.

Close
