# Supplementary material for: Whole-genome sequence of the oriental lung fluke Paragonimus westermani
Source: Gigascience. 2018 Dec 6;8(1):giy146. doi: 10.1093/gigascience/giy146 (PMC6329441; doi:10.1093/gigascience/giy146)
Supplement: Reviewer_1_Report_(Original_Submission) -- Rodrigo Baptista, Ph.D. — 7/11/2018 Reviewed [file giy146_reviewer_1_report_(original_submission).pdf]

Reviewer Report

Title: Whole-genome sequence of the oriental lung fluke Paragonimus westermani

Version: Original Submission **Date:** 7/11/2018

Reviewer name: Rodrigo Baptista, Ph.D.

Reviewer Comments to Author:

Oey et al. is well written and the presented data were well conducted investigating the first draft whole genome assembly of the oriental lung fluke Paragonimus westermani. No genome was available for this species before this present work showing that this is an important contribution to the field. The manuscript was concise, and the group did a nice work putting together a draft genome assembly of this such difficult highly repetitive genome.
However, this work is mainly focused in the genome assembly and could be strengthened with a few additional analysis/changes as follows:

Major

Title
- I suggest a small change in the manuscript title: "Draft Whole genome sequence of the oriental lung fluke…" or just "Whole genome sequence of the …". The term complete for nuclear genome sequence means that it is the final version (in chromosome level with no gaps), not the case here where the genome is still in 30,977 pieces, so complete should be not used here. The mitochondrial indeed looks complete.

Data Description

- The authors did not mention how they removed potential contamination or how they maintained the pathogen for the DNA extraction (Please add this information);
- Table 1 could be used as supplemental material;
- The assembly was performed by well-known genome assemblers, but there was any particular reason to not use any of the two most used PacBio assemblers (HGAP and CANU?);
- The authors choose to use for the Illumina assembly the ABYSS assembler. From my personal experience and from some colleagues there are several other assemblers that give a better job than ABYSS (Spades, MIRA, Velvet and SoapDenovo2). I know that it varies depending of the nature of the organism and sample used for the assay, but since the group used for the gapfilling step the soapDenovo gapcloser, I would like to see in the manuscript some information about why these pipelines were choosen beside others;
- Line 179 - REAPR typo. I would also suggest the authors to perform for this final polishing genome correction step Pilon or ICORN2 using the Illumina reads generated;
- Please add more information about the genome assembly statistics in table 2 (L50 and number of Ns), a quick run on QUAST should give you this information. And please explain if these gaps are just generated during the scaffolding by the mate pair evidence or it was also generated for unknown size gaps (100Ns). This information is really important to show that some regions could be missing in this draft genome assembly, so future studies could be aware of this fact;
- Line 250 - Since the ncRNA information was so important in the mitochondrial annotation, and the group already characterized the tRNAs, please add the method to predict these tRNAs (like tRNAscan) and also, I suggest adding an Aragorn or inferno ncRNA prediction run to improve even more the annotation;
- Line 258 - no problem with the methodology, but Cufflinks has a substitute, StringTie (Petera at el., 2015). It will do a much better job to assembly the transcriptome;
- Genome Comparison - I understand that this was not the focus of this manuscript, but sequence

identity besides important is a too general comparison method. I suggest add a orthology analysis and maybe generate a Circos synteny plot comparing the new genome with the most similar species available;
- Phylogeny - Add a Modeltest run to check if Jones-Taylor-Thornton (JTT) was the best substitution method to be used. For the ML analysis I suggest using PhyML instead of Phylip again, the software used is good but better and newer ones were developed;
- Bayesian method - MCMCTREE in PAML is good, but since Bayesian methods tend to vary, I suggest the group to run another test using the most known softwares (BEAST or mrBayes), to check if these mrca inferences are matching properly;

Figures
- Figure 1 - Doesn't need to be a main figure. Could be used as supplementary figure.
- Figure 2 B - These sequences could be mentioned in the text and added as supplementary file. You can name these repeats if needed in figure 2 A.
- Figure 3 - Figure is fine but needs to improve image quality. It is preferable to have a Venn diagram of the orthologs between these species.
- Add a circus synteny plot figure between the new genome and the closest species genome available.
- Figure 4 - (optional) Try to make the same figure using Figtree. They have a nicer way to show the median of the mrca on each node.

Minor

- Change the word faeces for stool. It's not wrong, but stool is more commonly used worldwide;
- Line 148 - Data Sequencing: add the Illumina Platform used in the data generation (example: HiSeq2000);
- Line 150 - Data Sequencing: add the PacBio Platform used in the data generation (example: PacBio Sequel or RSII);

Methods

Are the methods appropriate to the aims of the study, are they well described, and are necessary controls included? Choose an item.

Conclusions

Are the conclusions adequately supported by the data shown? Choose an item.

Reporting Standards

Does the manuscript adhere to the journal's guidelines on [minimum standards of reporting?] Choose an item.

Choose an item.

Statistics

Are you able to assess all statistics in the manuscript, including the appropriateness of statistical tests used? Choose an item.

Quality of Written English

Please indicate the quality of language in the manuscript: Choose an item.

Declaration of Competing Interests

Please complete a declaration of competing interests, considering the following questions:

- Have you in the past five years received reimbursements, fees, funding, or salary from an organisation that may in any way gain or lose financially from the publication of this manuscript, either now or in the future?
- Do you hold any stocks or shares in an organisation that may in any way gain or lose financially from the publication of this manuscript, either now or in the future?
- Do you hold or are you currently applying for any patents relating to the content of the manuscript?
- Have you received reimbursements, fees, funding, or salary from an organization that holds or has applied for patents relating to the content of the manuscript?
- Do you have any other financial competing interests?
- Do you have any non-financial competing interests in relation to this paper?

If you can answer no to all of the above, write 'I declare that I have no competing interests' below. If your reply is yes to any, please give details below.

I declare that I have no competing interests

I agree to the open peer review policy of the journal. I understand that my name will be included on my report to the authors and, if the manuscript is accepted for publication, my named report including any attachments I upload will be posted on the website along with the authors' responses. I agree for my report to be made available under an Open Access Creative Commons CC-BY license (http://creativecommons.org/licenses/by/4.0/). I understand that any comments which I do not wish to be included in my named report can be included as confidential comments to the editors, which will not be published.

I agree to the open peer review policy of the journal

To further support our reviewers, we have joined with Publons, where you can gain additional credit to further highlight your hard work (see: https://publons.com/journal/530/gigascience). On publication of this paper, your review will be automatically added to Publons, you can then choose whether or not to claim your Publons credit. I understand this statement.

Yes Choose an item.
