# Supplementary material for: Whole-genome sequence of the oriental lung fluke Paragonimus westermani
Source: Gigascience. 2018 Dec 6;8(1):giy146. doi: 10.1093/gigascience/giy146 (PMC6329441; doi:10.1093/gigascience/giy146)
Supplement: Reviewer_2_Report_(Original_Submission) -- Krystyna Cwiklinski — 7/16/2018 Reviewed [file giy146_reviewer_2_report_(original_submission).pdf]

Reviewer Report

Title: **Whole-genome sequence of the oriental lung fluke Paragonimus westermani**

Version: **Original Submission** **Date:** 7/16/2018

Reviewer name: **Krystyna Cwiklinski**

Reviewer Comments to Author:

The manuscript by Oey and colleagues reports the first draft genome for the lung fluke parasite Paragonimus westermani. This study improves on the limited sequence data available for this parasite and complements the vast amount of omics data now currently available for trematode parasite species.
The manuscript is currently written in my opinion as a data note rather than a research type manuscript. If this is the intention this should be made clearer by the authors as part of their submission. If the manuscript is intended to be submitted as a research paper, the authors should expand on their discussion and conclusions of their data.
The sequencing and assembly of the genome is technically sound but the manuscript currently lacks any interpretation of the genome data, particularly the functional annotation. I believe the manuscript is suitable for publication in GigaScience, following the below points that require clarification and/or comments by the authors.

1. Abstract, line 85 and Data description, line 157: The authors computationally determined the estimated size of the P.westermani genome, prior to assembly of the raw reads. The computationally determined estimated size was slightly larger than the assembled genome size. The authors should comment on the size difference. In addition, the authors interchange throughout the manuscript whether they compare the estimated size or the assembled genome size with other known published trematode genomes. Until it can be shown that the genome of P.westermani is actually 1.1 Gb, the authors should only refer to the assembled genome size particularly in the section around line 157, as these published trematode genomes describe only the assembled genome sizes.
2. Line 144 - at what point of infection were the parasites recovered - specifically how old were the parasites?
3. Lines 144-146 -Further information is required regarding the methodology of genomic DNA extraction. Was the extraction carried out on individual worms and then combined or were the worms combined for extraction? Was the genomic DNA quality checked?
4. Line 150 - can the authors confirm that the PacBio sequencing was performed on the same sample of genomic DNA?
5. Line 255 - the authors should mention that the RNAseq data was from adult parasites only, not the various different lifecycle stages.
6. Line 221 - Related to point 3, as the authors extracted DNA from 50 individual worms, did they check the level of polymorphism at the individual worm level for this region?
7. Line 272-273 - If the authors are submitting a research themed manuscript, they could include some further discussion of the predicted protein coding genes, particularly those predicted proteins that have inferred homologs in other trematodes (Fig 3A) and the Paragonimus-specific predicted proteins.
8. Figure 3A - the venn diagram is currently difficult to interpret, particularly given its current small size as a multi-panel figure. I suggest amending this figure to a classical venn diagram or an Upset plot.
9. The authors should include supplemental data detailing the functional annotation particularly the analysis of the functional domains, transmembrane domains and signal peptides, as well the data relating to the single copy predicted proteins used for the phylogenetic analysis.
10. Minor corrections:

a. line 118 - develop into sporocysts
b. line 161 - 1.3 Gb

c. line 204, 291, 293 - BLAST
d. line 281 - predicted proteome

Methods

Are the methods appropriate to the aims of the study, are they well described, and are necessary controls included? Choose an item.

Conclusions

Are the conclusions adequately supported by the data shown? Choose an item.

Reporting Standards

Does the manuscript adhere to the journal's guidelines on minimum standards of reporting? Choose an item.

Choose an item.

Statistics

Are you able to assess all statistics in the manuscript, including the appropriateness of statistical tests used? Choose an item.

Quality of Written English

Please indicate the quality of language in the manuscript: Choose an item.

Declaration of Competing Interests

Please complete a declaration of competing interests, considering the following questions:

- Have you in the past five years received reimbursements, fees, funding, or salary from an organisation that may in any way gain or lose financially from the publication of this manuscript, either now or in the future?
- Do you hold any stocks or shares in an organisation that may in any way gain or lose financially from the publication of this manuscript, either now or in the future?
- Do you hold or are you currently applying for any patents relating to the content of the manuscript?
- Have you received reimbursements, fees, funding, or salary from an organization that holds or has applied for patents relating to the content of the manuscript?
- Do you have any other financial competing interests?
- Do you have any non-financial competing interests in relation to this paper?

If you can answer no to all of the above, write 'I declare that I have no competing interests' below. If your reply is yes to any, please give details below.

I declare that I have no competing interests

I agree to the open peer review policy of the journal. I understand that my name will be included on my report to the authors and, if the manuscript is accepted for publication, my named report including any attachments I upload will be posted on the website along with the authors' responses. I agree for my report to be made available under an Open Access Creative Commons CC-BY license (http://creativecommons.org/licenses/by/4.0/). I understand that any comments which I do not wish to be included in my named report can be included as confidential comments to the editors, which will not be published.

I agree to the open peer review policy of the journal

To further support our reviewers, we have joined with Publons, where you can gain additional credit to further highlight your hard work (see: https://publons.com/journal/530/gigascience). On publication of this paper, your review will be automatically added to Publons, you can then choose whether or not to claim your Publons credit. I understand this statement.

Yes Choose an item.
